# Biomass-to-biomass strategy: sulphonic acid functionalized covalent organic frameworks for metal-free esterification of biomass-derived acids

**DOI:** 10.1039/d6ra06829d

**Published:** 2026-10-07

**Authors:** Shradhanjali Samal, Muskan Memon, Entesar Alhousin, Soyeb Pathan, Sonal Thakore

**Affiliations:** a Department of Chemistry, Faculty of Science, The Maharaja Sayajirao University of Baroda Vadodara 390 002 India sonal.thakore-chem@msubaroda.ac.in; b Research and Development Cell (RDC), Parul University Vadodara 391760 India; c Department of Chemistry, Parul Institute of Applied Sciences, Parul University Waghodia Vadodara Gujarat 391760 India khan_9751@yahoo.com

## Abstract

A sulphonic acid functionalized Covalent Organic Framework (COF–SO_3_H) was synthesized *via* an ultrasound-assisted aqueous-phase Schiff base condensation, providing a green and efficient route to incorporate Brønsted acidic sites directly into the framework. Characterisation techniques showed that the resulting material exhibits a partially crystalline, hierarchically porous structure with a surface area of 185 m^2^ g^−1^. DFT calculations performed using B3LYP reveal a well-defined hexagonal pore topology with uniformly distributed –SO_3_H groups along the framework edges. The catalytic activity of COF–SO_3_H was evaluated for the esterification of biomass-derived molecules, levulinic acid (LA) and succinic acid (SA), with butyl alcohol. Under optimized conditions, the metal-free catalyst afforded excellent conversion (96%) of LA with 99.7% selectivity toward *n*-butyl levulinate, while SA esterification showed tunable selectivity between mono- and diesters depending on reaction parameters. The catalytic performance is attributed to the accessible –SO_3_H sites and efficient mass transport within the porous framework. The catalyst demonstrated good stability and recyclability over multiple cycles with minimal loss of activity. The scope of the esterification catalysis could also be extended to various other alcohols, including secondary alcohols and cyclohexanol. It was observed that the chain length and structure of alcohols have significant influence on conversion and selectivity. This study establishes the potential utilization of –SO_3_H functionalized COFs as sustainable metal-free heterogeneous acid catalysts for the valorization of biomass-derived acids into value-added esters.

## Abstract 2

A metal-free sulfonic acid functionalized COF serves as a tunable heterogeneous acid catalyst for selective esterification of bio-based levulinic and succinic acids, yielding 99.7% *n*-butyl levulinate and tailored succinate esters.

## Graphical abstract

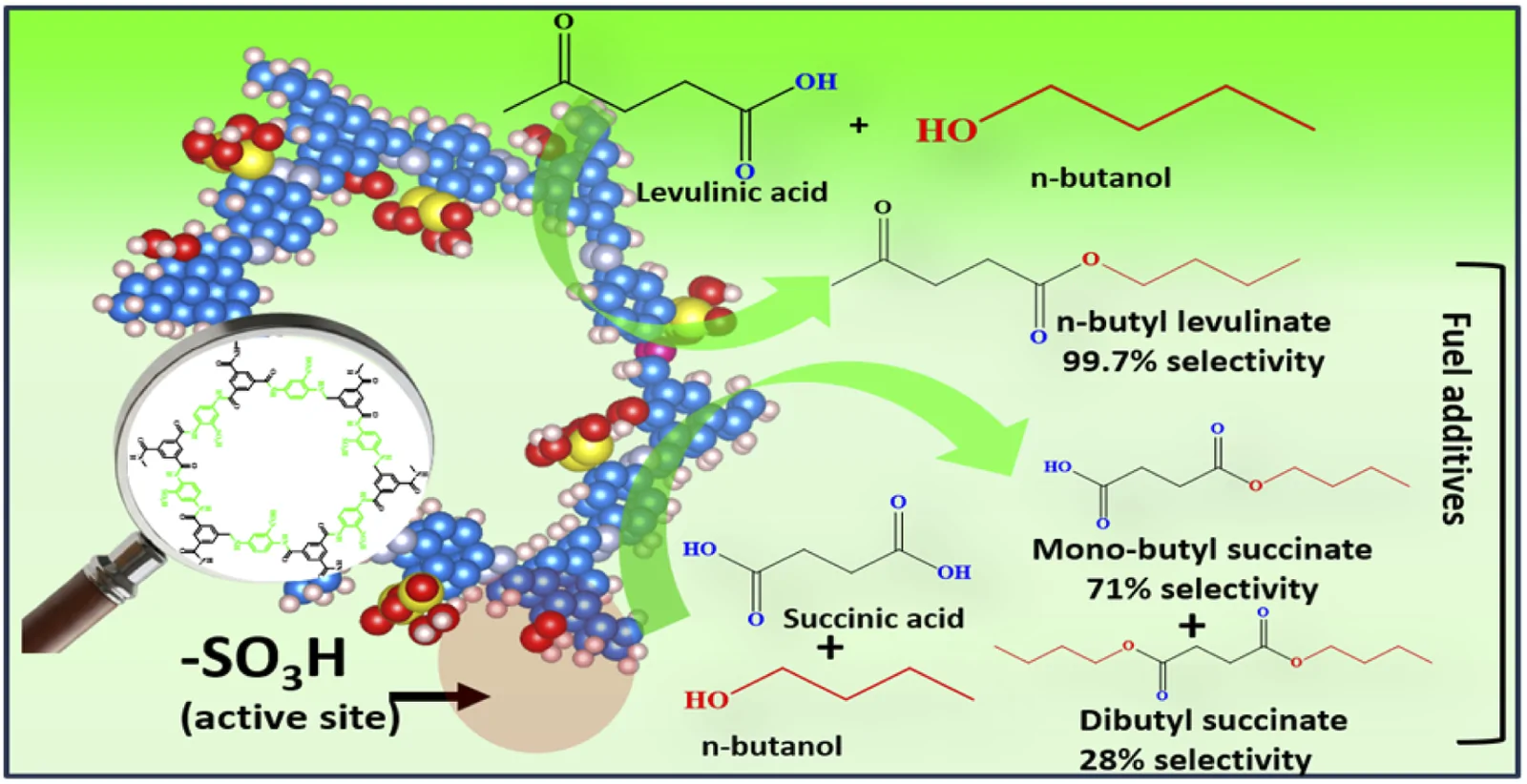

## Introduction

The finite availability of fossil fuels, coupled with escalating environmental concerns, has intensified efforts toward identifying renewable alternatives.^1^ Biomass has emerged as a promising feedstock owing to its potential to generate a wide spectrum of value-added chemicals, promising fuel additives, biofuels, and commodity materials within the framework of biorefinery concepts.^2^ Among the biomass-derived platform molecules prioritized by the U.S. Department of Energy, levulinic acid (LA) and succinic acid (SA) hold prominent positions due to their versatile reactivity and broad industrial applicability.^3^ The efficient catalytic conversion of such bio platform molecules through routes including esterification,^4^ hydrogenation,^5^ and reductive amination^6^ mitigates dependence on fossil resources. This establishes a sustainable pathway for the production of high-value chemicals and fuels from renewable biomass.

Despite these advances, current esterification catalysts still face significant limitations concerning catalyst stability, recyclability, water management, and feedstock quality.^7,8^ Both homogeneous and heterogeneous systems are prone to deactivation due to impurities in bio-derived feedstocks such as water, metal cations, and organic residues that poison active sites or cause leaching. The *in situ* formation of water during esterification shifts the reaction equilibrium backward, lowering yields, while the poor solubility of crystalline SA in alcohols complicates continuous operation and necessitates additional pre-treatment steps.^9^ Achieving high conversion typically requires excess anhydrous alcohol and efficient water removal methods to drive the reaction forward. Similarly, for LA, the use of mineral acids and ion-exchange resins often results in low conversions under mild conditions, especially with bulky alcohols.^10^ Reaction rates are further influenced by steric and electronic effects of alcohols, while impurities limit scalability to batch or semi-batch modes. Therefore, the development of water-tolerant, recyclable, and selective catalytic systems is essential to achieve sustainable and industrially viable routes for producing bio-based esters.

In recent years, functionalized porous materials have garnered significant research attention owing to their exceptional physicochemical properties, including very high surface areas and the ability to be chemically tailored through the incorporation of reactive functional groups.^11–13^ Moreover, their tunable framework composition, geometry, and pore dimensions, determined by the symmetry and connectivity of the building monomers, make them highly versatile for a wide range of applications. Among various inorganic and organic porous systems, Covalent Organic Frameworks (COFs) represent a particularly promising class that has been extensively explored over the past few decades. COFs are crystalline, fully designable porous materials composed of organic building blocks linked by robust covalent bonds, forming ordered and periodic pore networks. Their well-defined structures and functional tunability enable COFs to serve in diverse applications such as heterogeneous catalysis,^14^ metal-ion sensing, adsorption,^15^ drug delivery, and CO_2_ capture.^16^ These multifunctional capabilities arise from the π-conjugated organic skeletons and highly ordered atomic layers within COFs, which provide confined spaces for effective interactions with photons, excitons, electrons, holes, and guest species.

Recent progress in catalyst design centres on new materials that can overcome major limitations in catalysis. Although heterogeneous catalysts have been reported for esterification of levulinic and succinic acids, limitations in active-site accessibility and catalyst design remain.^17,18^ A notable development is the creation of covalent organic frameworks (COFs), which are remarkable porous solids with exceptionally high surface areas, adjustable pore sizes, and customizable chemical functionalities.^19,20^ COFs are formed by linking organic molecules through sturdy covalent bonds, resulting in highly structured, crystalline frameworks that can be engineered for specific tasks.^11,21^ These distinctive features make COFs ideal candidates for catalytic applications, as they allow easy access to catalytic sites while enabling precise modification of the chemical environment within the framework for targeted reactivity. The use of SO_3_H-functional group^22^ in COF offers the combined advantages of strong Brønsted acidity, a porous heterogeneous environment, and facile catalyst separation, while avoiding the use of metal-based active sites. Thus, the incorporation of –SO_3_H group in the framework has been identified as a key strategy for enhancing catalytic performance in biomass conversion. Previously, sulphonated-COFs have been used for the dehydration of fructose to 5-hydroxymethylfurfural.^23^

A sulphonic acid functionalized COF was synthesized under aqueous conditions and used for the esterification of SA and LA under mild conditions with *n*-butanol. Reaction conditions like catalyst amount, temperature, mole ratio of LA and SA with *n*-butanol were screened to obtain the maximum yield. Various alcohols were employed to obtain various succinic and levulinic acid esters. The study demonstrates deep insights for developing a heterogeneous acid catalyst for efficient esterification reactions for converting lignocellulosic biomass-derived molecules into alkyl levulinates and alkyl succinates. To the best of our knowledge, there are no reports on the synthesis of sulphonated COF for esterification of these bioplatform molecules.

## Materials and methods

### Materials

Succinic acid, *n*-propanol (>99%, AR grade), iso-propanol (99%), *n*-butanol (99%), butan-2-ol (99%), iso-butanol (99%), *n*-hexanol (98%), cyclohexanol (99%), glycerol (99.5%), and furfuryl alcohol (98%) were purchased from Loba Chemie Pvt. Ltd, while levulinic acid (>97%) and methanol (HPLC grade) were purchased from Himedia.

### Synthesis of sulphonic acid functionalized COF (COF–SO_3_H)

The COF–SO_3_H was tailored by Schiff base condensation to form imine linkage between the monomers. Briefly, 2-hydroxybenzene-1,3,5-tricarbaldehyde (50 mg, 0.28 mmol) and 1,4-phenylenediamine-2-sulphonic acid (79 mg, 0.42 mmol) were added in a ratio of 3 : 2 in 6 M acetic acid solution (10 mL) in a round bottom flask (Scheme 1). The reaction mixture was degassed by purging nitrogen for 15 min and stirred at room temperature (28 °C) for 2 h to yield a dark violet coloured product. The product was filtered using Whatman filter paper no. 41 and washed with deionised water, acetone, methanol and dichloromethane to remove impurities. The obtained product (120 mg, 93% yield) was vacuum-dried at 120 °C for 2 h.

**Scheme 1:**
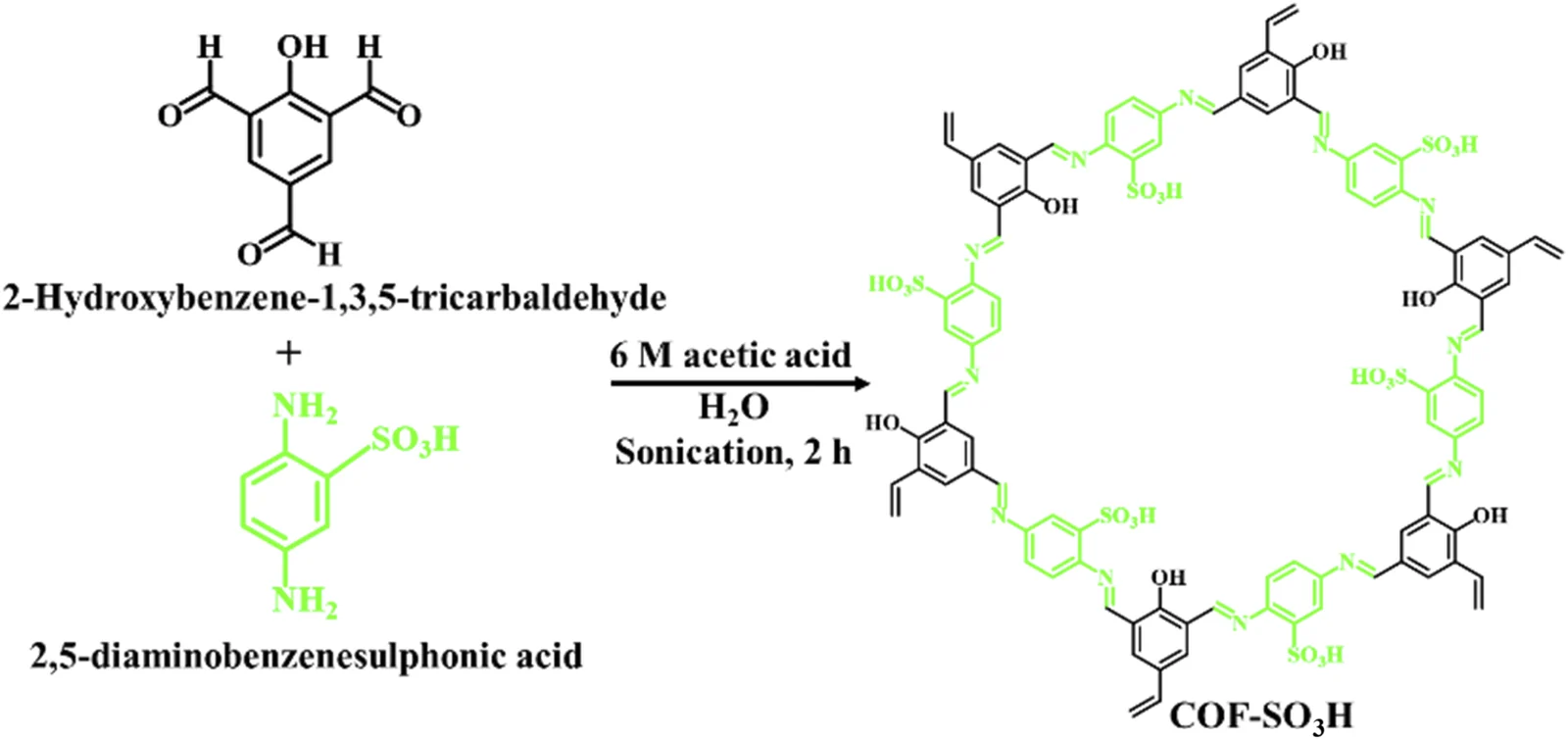
Synthesis of sulphonic acid functionalized COF (COF–SO_3_H).

### Acidity determination

A UV-visible spectrophotometer was employed to evaluate the relative acidity of the different sulfonated COFs, utilizing 4-nitroaniline as a basic indicator [I]. For this purpose, an aqueous solution was prepared by combining 4-nitroaniline (5 mg L^−1^ concentration, p*K*_a_ of 0.99) with samples at a 5 mg L^−1^ concentration. The UV absorbance peak was detected at 382 nm wavelength.^23,24^ It was noted that increased catalyst acidity corresponded to decreased indicator absorbance. The protonated indicator form, [HI]^+^, showed minimal molar absorptivity. H°, the Hammett function, for each catalyst, was determined by determining the absorption difference between I and HI^+^ concentrations using eqn (1)

1 *H*° = p*K*_a_ + log[I]/[HI]^+^

The p*K*_a_ value in this equation indicates the acidity of the basic indicator when dissolved in water. Additionally, [I] and [HI]^+^ represent the concentrations of the indicator and its protonated form, respectively. Moreover, acid–base titration was considered to measure the acid amounts of COF–SO_3_H; 50 mg of each sample was stirred in saturated NaCl solution (15 mL) for 6 h at ambient temperature. Afterward, the supernatant was collected by separating solid from the mixture and was titrated with NaOH solution (0.01 M) using the phenolphthalein indicator.

### Characterization

Powder X-ray diffraction (PXRD) analysis of COF–SO_3_H was carried out using a Bruker D8 Discover diffractometer with an angular scanning step size of 0.02 s^−1^. The diffraction patterns were obtained after concentrating the COF sample and placing it on a clean glass plate. The structural characteristics were obtained through solid-state CP-MAS NMR spectroscopy using an ECZR series 600 MHz NMR spectrometer (JEOL, Japan) equipped with a 3.2 mm rotor and a fast MAS probe operating at a spinning speed of 20 kHz. The spectra were processed using Delta NMR Data Processing Software. The FT-IR spectra were recorded on a Bruker (Alpha II) instrument (Karlsruhe, Germany) in the range of 4000 to 500 cm^−1^. The morphology was investigated using Field Emission Gun Scanning Electron Microscopy (FEG-SEM). Images were obtained with a JEOL JEM-2100F electron microscope operated at an accelerating voltage of 300 kV. To enhance sample conductivity during SEM analysis, a thin platinum coating was applied to the surface under vacuum prior to imaging. The Brunauer–Emmett–Teller (BET) surface area was determined using a Micromeritics ASAP 2020 surface area and pore size analyzer from N_2_ adsorption–desorption isotherms measured at 77 K, employing the Barrett–Joyner–Halenda (BJH) method. Prior to analysis, the samples were degassed at 120 °C under vacuum for 12 hours. The temperature and vacuum conditions were stabilized for an additional 7 hours before measurements. Thermogravimetric analysis (TGA) was performed using a TGA 2950 instrument in the temperature range of 25–750 °C under a nitrogen flow of 30 mL min^−1^, with a heating rate of 10 °C min^−1^. X-ray photoelectron spectroscopy (XPS) was conducted using an ESCA system (Omicron Nanotechnology), employing Al Kα radiation (1486 eV) under ultra-high vacuum (UHV) conditions. Density Functional Theory calculations were performed using B3LYP/6-31G (Becke, 3-parameter, Lee–Yang–Parr) using Gaussian software.

### Catalytic evaluation

The reactions were conducted in a 50 mL round-bottom flask with a single neck fitted with an air condenser and guard tube. The reaction mixture contains 1.18 g of SA (10 mmol), 3.6 mL of *n*-butanol (40 mmol) and 40 mg of catalyst amount and was allowed to stir at desired temperature (90 °C) and time (5 h). The same procedure was carried out for levulinic acid esterification which contains LA (10 mmol), *n*-butanol (30 mmol) and 40 mg of catalyst amount were allowed to stir at temperature (90 °C) and time (8 h). Once the reaction was completed, the mixture was cooled to room temperature, diluted using 10 mL of methanol, and separated using a simple centrifugation step and was analyzed using gas chromatography (Shimadzu-2014C, SH-5 capillary column with an internal diameter of 0.25 mm, length: 30 m). The esterification product of SA and LA were identified using GC-MS (Shimadzu GCMS-QP2020 NX). The % conversion (based on SA and LA) and selectivity were calculated from the given formulae:
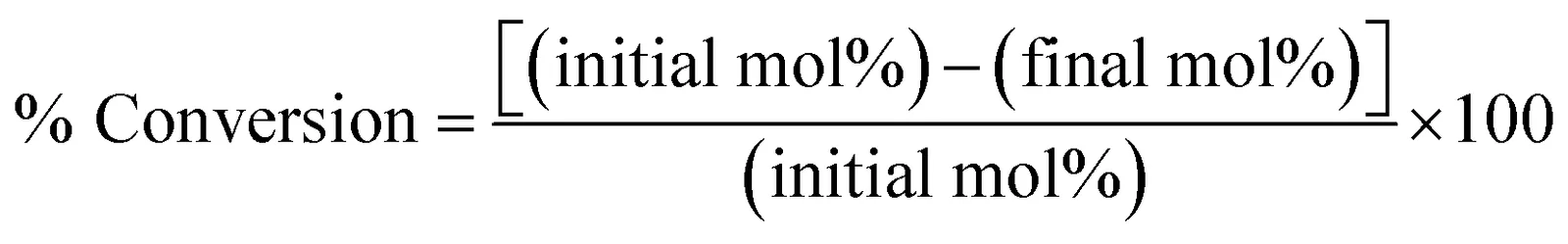

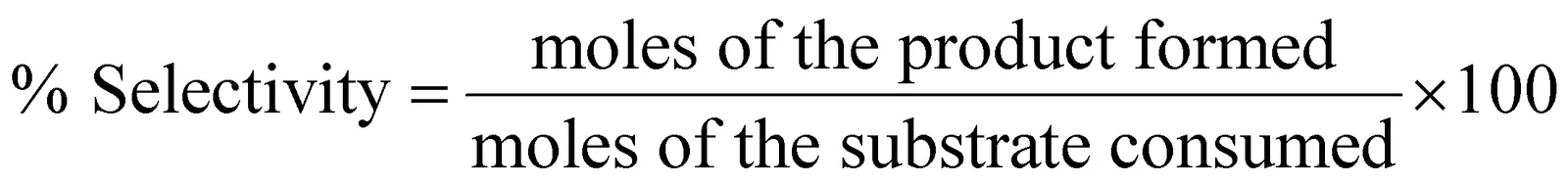


## Results and discussion

### Material characterization

Herein, we report the synthesis of COF–SO_3_H in aqueous medium with acetic acid as catalyst. To avoid the need for post-synthetic modifications in COFs, monomers containing –SO_3_H groups were selected during the initial framework design. This strategy streamlines the synthesis by incorporating the desired functionality directly into the COF structure from the outset. The synthesis was accomplished through the ultrasound assisted condensation of 2-hydroxy 1,3,5-triformyl benzene with 1,4-phenylenediamine 2-sulphonic acid in an aqueous solvent system. The use of ultrasonication facilitated efficient dispersion and activation of the reactants, promoting rapid nucleation and uniform framework growth under mild conditions. This green and solvent-safe approach not only enhanced reaction kinetics but also enabled the formation of a well-ordered crystalline COF structure with high yield and purity. Compared to previously reported synthesis methods, the present approach is more environmentally friendly and sustainable, utilizing water as the solvent and requiring significantly less reaction time. The acquired COF is insoluble in solvents like methanol, ethanol, acetone, dichloromethane and tetrahydrofuran.

The relative acidity of the sulfonated COF was first examined using the Hammett acidity function to determine the extent of sulfonic acid incorporation. A measured Hammett value (*H*_o_) of 1.56 reflects the presence of highly active Brønsted acid sites in the framework. As per CHNS analysis, 2.28 g catalyst contains 7.96% sulphur, which corresponds to 5.69 mmol g^−1^ acidic strength.

### Optimised structure and MEP plot of COF–SO_3_H

The geometry optimized structure of the sulfonated covalent organic framework (COF–SO_3_H), obtained using the B3LYP functional, reveals a well-defined hexagonal pore topology with uniformly distributed –SO_3_H groups along the framework edges (Fig. 1A). The optimized conformation shows that the aromatic linkers and imine (–C=N–) bonds are planar, promoting extended π-conjugation and structural stability. The –SO_3_H groups are oriented toward the inner pore surface, facilitating accessible Brønsted acidic sites for catalytic interactions. The optimized bond lengths and angles around the sulphur and oxygen atoms remain consistent with typical sulfonic acid geometry (S–O = 1.45 Å, S=O = 1.42 Å), confirming the structural integrity of the functional group. The framework maintains a balanced spatial arrangement with minimal steric strain, supporting efficient charge distribution and pore accessibility.

**Fig. 1:**
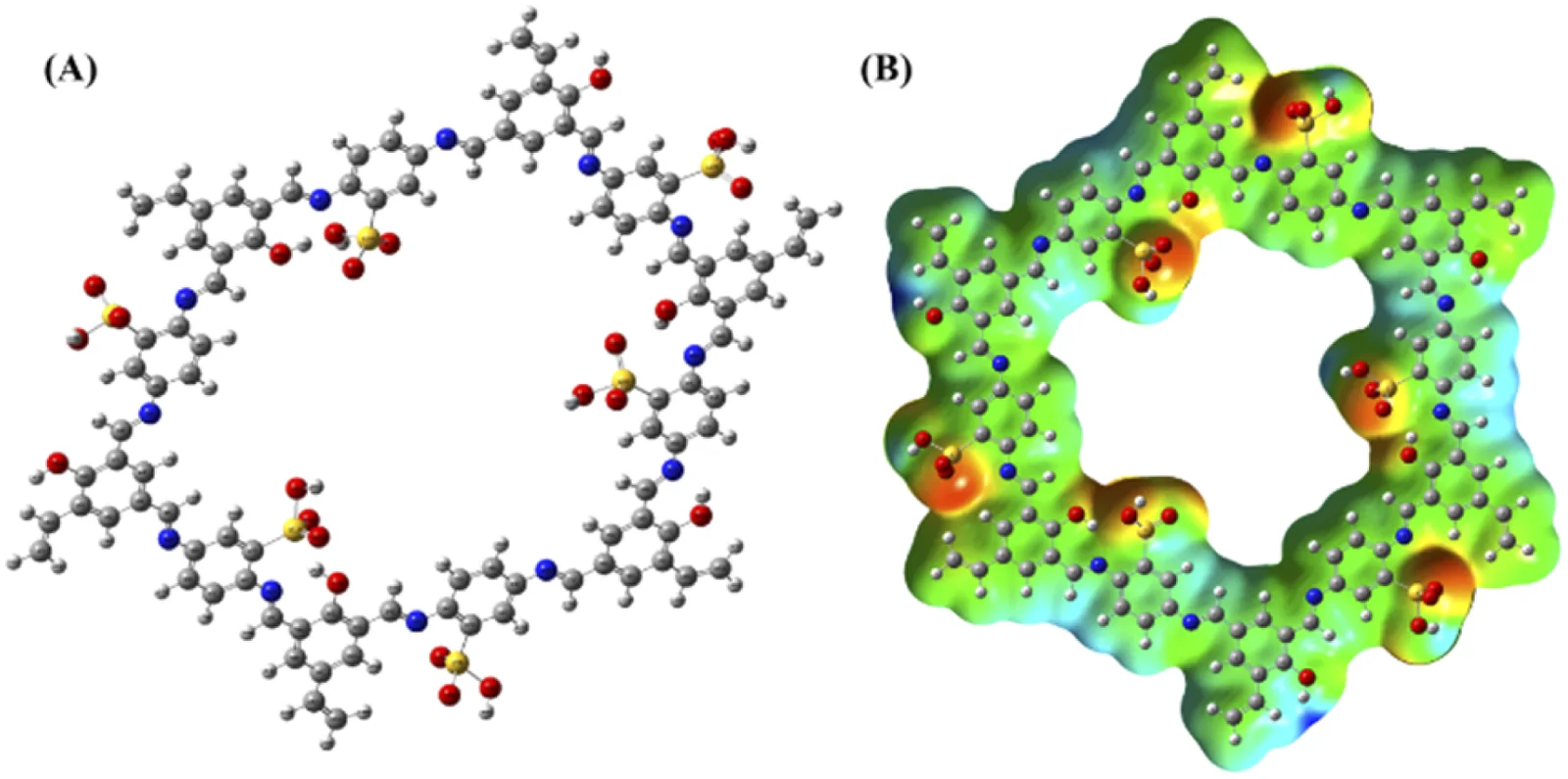
(A) Optimised structure of COF–SO_3_H (B) MEP diagram.

The molecular electrostatic potential (MEP) map of the COF–SO_3_H provides valuable insight into its charge distribution and potential reactive sites responsible for catalytic activity in esterification reactions (Fig. 1B). The MEP surface reveals a distinct polarization pattern, where the oxygen atoms of the –SO_3_H and –OH groups exhibit intense red regions, corresponding to areas of high negative electrostatic potential. These electron rich zones indicate strong Lewis basic or nucleophilic sites that can effectively interact with electrophilic centres of reactant molecules such as levulinic and succinic acid. In contrast, the blue regions localized around the hydrogen atoms of the sulfonic acid moieties represent positively charged acidic domains, capable of proton donation and substrate activation. The sulphur centres display moderate potential regions, reflecting their role in delocalizing electron density within the –SO_3_H groups, thereby stabilizing the overall charge distribution. The green areas spread along the aromatic backbone correspond to relatively neutral π-conjugated domains that maintain structural stability and facilitate charge transfer across the framework. The strong electronic polarization and acidity of the COF–SO_3_H framework is efficient for the esterification of levulinic and succinic acid with butanol.

The FTIR spectrum of the COF–SO_3_H exhibits distinct absorption bands confirming the successful formation of the imine-linked framework and incorporation of –SO_3_H groups (Fig. 2A). A broad band centred at 3415 cm^−1^ corresponds to the O–H stretching vibration of phenolic and sulfonic acid groups, indicating the presence of acidic functionalities. The sharp band at 1624 cm^−1^ is assigned to the C=N stretching vibration, confirming the formation of imine linkages through condensation of amine and aldehyde monomers.^14^ The characteristic peaks observed at 1375 cm^−1^ and 1182 cm^−1^ are attributed to the asymmetric and symmetric S=O stretching vibrations of the sulfonic acid group, respectively, confirming successful sulfonation of the aromatic framework.^23^ The absorption at 1464 cm^−1^ corresponds to aromatic C=C stretching, supporting the presence of a conjugated benzene ring system. The absence of any carbonyl peak near 1700 cm^−1^ further verifies complete conversion of the aldehyde precursors. These spectral features collectively affirm the formation of a chemically robust, sulfonic acid functionalized COF with well-defined acidic sites suitable for catalytic applications.

**Fig. 2:**
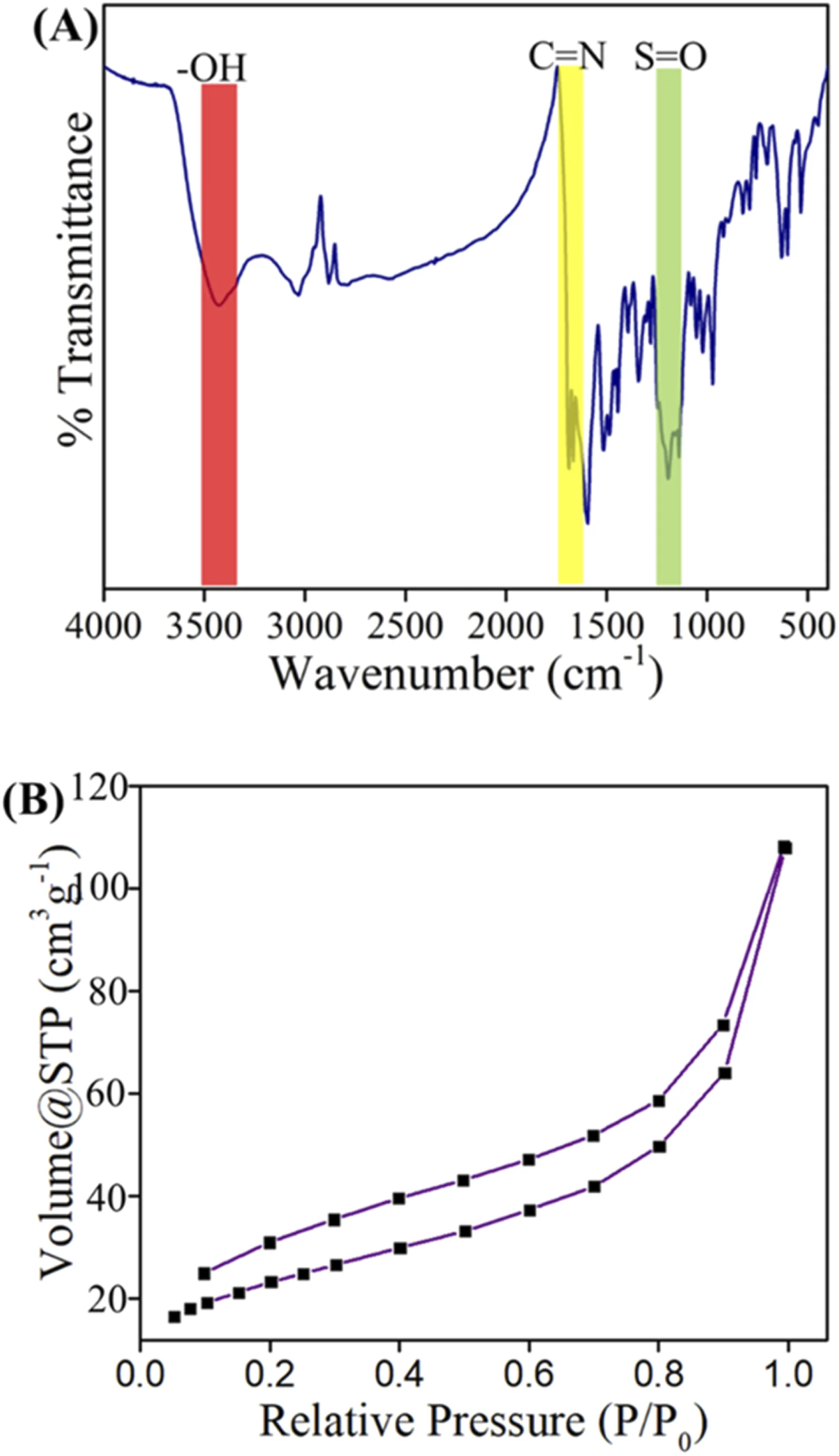
(A) FTIR and (B) BET adsorption–desorption isotherm of COF–SO_3_H.

The nitrogen adsorption–desorption isotherm of the COF–SO_3_H displays a type IV isotherm with a distinct hysteresis loop, confirming its mesoporous nature (Fig. 2B).^23^ The COF exhibits a BET surface area of 185 m^2^ g^−1^ and a total pore volume of 0.28 cm^3^ g^−1^, corresponding to an average pore diameter of approximately 6.1 nm.^25^ The gradual adsorption at low relative pressures indicates the presence of micropores, while the sharp uptake at higher pressures reflects capillary condensation within mesopores. The hysteresis pattern is H3 type which is suggested by the slit-shaped pores, typical of layered COF structures. These textural properties confirm the formation of a hierarchically porous framework that facilitates efficient diffusion and accessibility of –SO_3_H active sites, making the material promising for catalytic and adsorption-based applications.

The solid-state NMR spectrum confirms the successful formation of the sulfonated COF–SO_3_H (Fig. 3). The broad resonance observed in the aromatic region between 110–160 ppm corresponds to the carbon atoms of the aromatic rings, indicating a well-extended conjugated framework.^24^ A distinct signal around 155–165 ppm is attributed to the C=N linkage, confirming the formation of imine bonds between the amine and aldehyde precursors, a key feature of COF construction. Additionally, the appearance of downfield peaks near 145–155 ppm suggests the presence of C–SO_3_H and C–OH carbons, verifying the incorporation of sulfonic acid groups within the COF structure. The absence of any aldehydic carbon peak between 190–210 ppm further supports complete condensation, establishing the formation of a chemically stable, highly functionalized COF with acidic sites suitable for catalytic applications.

**Fig. 3:**
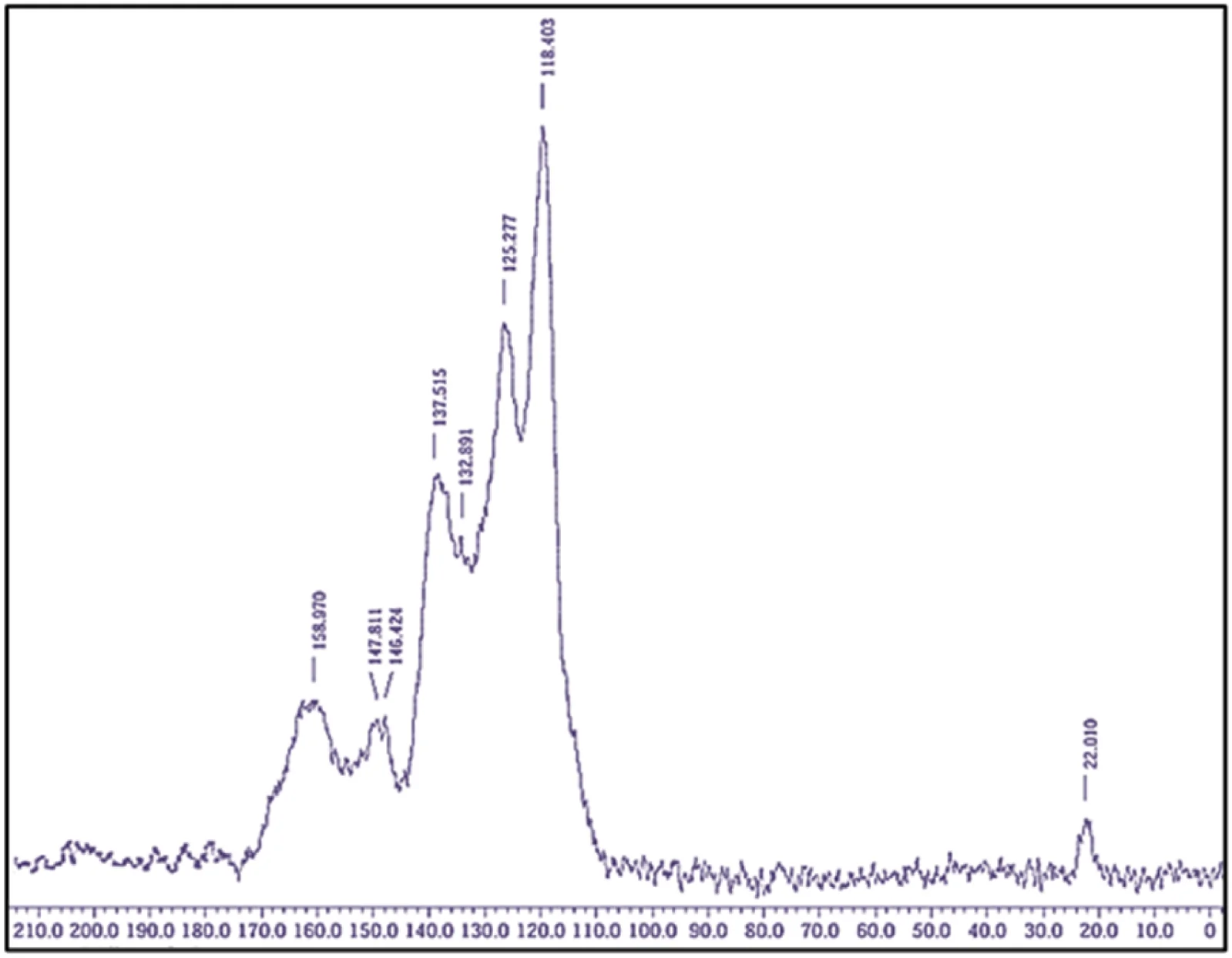
^13^C solid state NMR of COF–SO_3_H.

SEM analysis at different morphology of COF possesses a characteristic layered and sheet-like morphology with rough, network -like and interconnected porous domains (Fig. 4A and B). The material is composed of aggregated nanosheets that fold, overlap, and stack irregularly, giving rise to a textured architecture with numerous edges and void spaces. These loosely arranged sheets create a network of accessible pores and channels throughout the structure, reflecting the inherently porous nature. The EDS shows the elemental composition of COF (Fig. 4C).

**Fig. 4:**
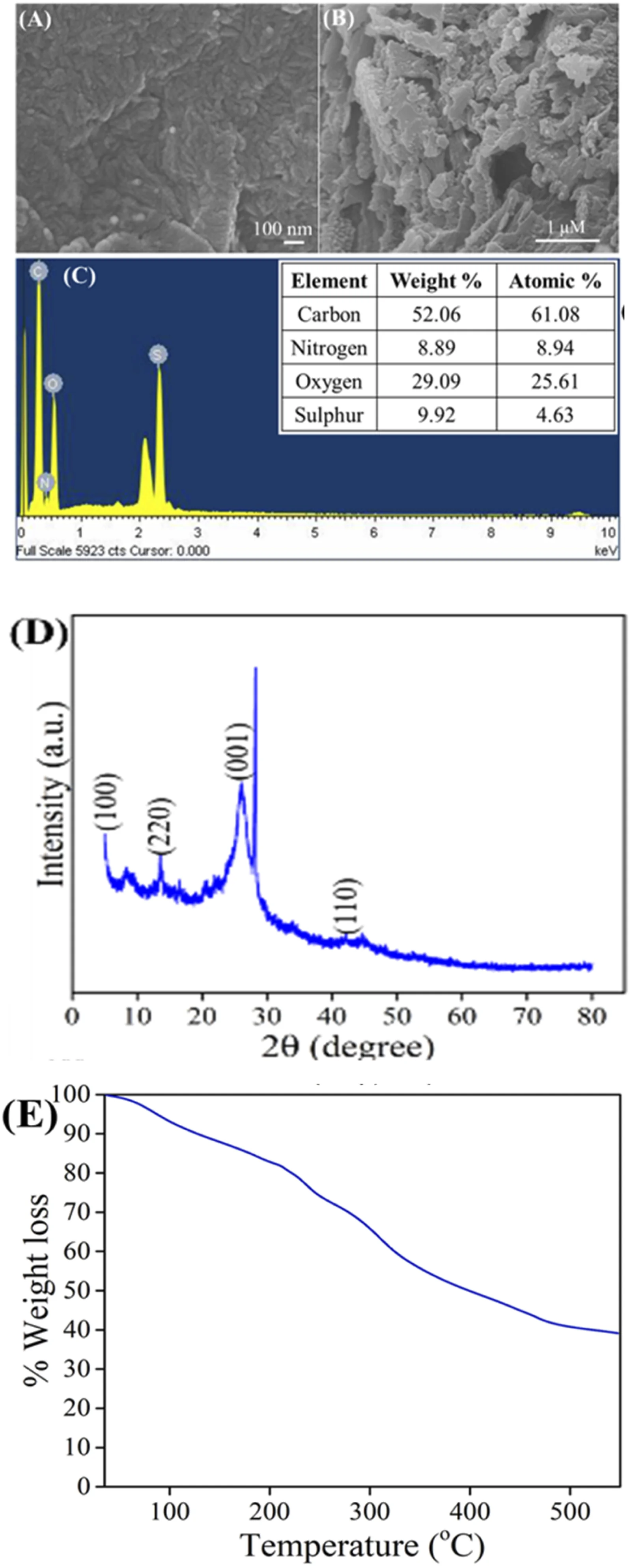
(A and B) FESEM at different magnification (C) EDS (D) XRD (E) TGA of COF–SO_3_H.

The powder X-ray diffraction (PXRD) pattern of the sulfonic acid-functionalized COF confirms the formation of a partially crystalline framework (Fig. 4D). The diffraction peaks observed at 2*θ* = 4.8°, 11.9, 27.5°, and 43.2° correspond to the (100), (220), (001), and (110) planes, respectively, characteristic of an ordered layered COF structure. The intense peak around 27.5° is attributed to π–π stacking between adjacent aromatic layers, indicating the presence of interlayer ordering. The nature of the diffraction peaks suggests a crystalline structure, which indicates a long-range ordered structure for the functionalized COF. The thermogravimetric analysis (TGA) suggests that the COF is stable up to 250 °C. Since COF was synthesized in an acetic acid solution, the initial mass loss is due to trapped moisture of 8%. Further, a decrease in mass of about 12% is observed between 150 °C to 250 °C (Fig. 4E).

To elucidate the surface elemental composition and chemical bonding states of the synthesized COF–SO_3_H, high-resolution X-ray photoelectron spectroscopy (XPS) analysis was conducted (Fig. 5). As depicted in the survey spectra (Fig. S1), the framework displays characteristic signals corresponding to carbon, nitrogen, oxygen, and sulfur environments, which is consistent with the structure. The high-resolution C 1s spectrum is deconvoluted into two prominent sub-peaks. The major peak centered at 284.6 eV is assigned to –C=C of the aromatic backbones and the pendant vinyl moiety –CH=CH_2_. The secondary peak located at 286.7 eV corresponds to the carbon atoms involved in the –C=N– linkages as well as phenolic C–O bonds within the aldehyde building block.^20^ The successful Schiff-base condensation is further validated by the N 1s spectrum, which exhibits a symmetric single peak centered at 400.1 eV.^26^ This signal is exclusively attributed to the imine nitrogen confirming complete transformation of the primary amine precursors into the covalent extended network without residual unreacted amine species. The peak at 531.0 eV is associated with the phenolic oxygen (C–O) and partial contribution from the sulfonate S=O oxygen atoms. The peak at higher binding energy (532.5 eV) corresponds to the hydroxyl oxygen (O–H) of the pendant sulfonic acid groups (–SO_3_H) alongside surface-adsorbed moisture. The binding energies at 170.1 and 168.6 eV were assigned to S 2p_1/2_ and S 2p_3/2_ respectively, indicating that sulfur exists exclusively as –SO_3_H groups.^27^

**Fig. 5:**
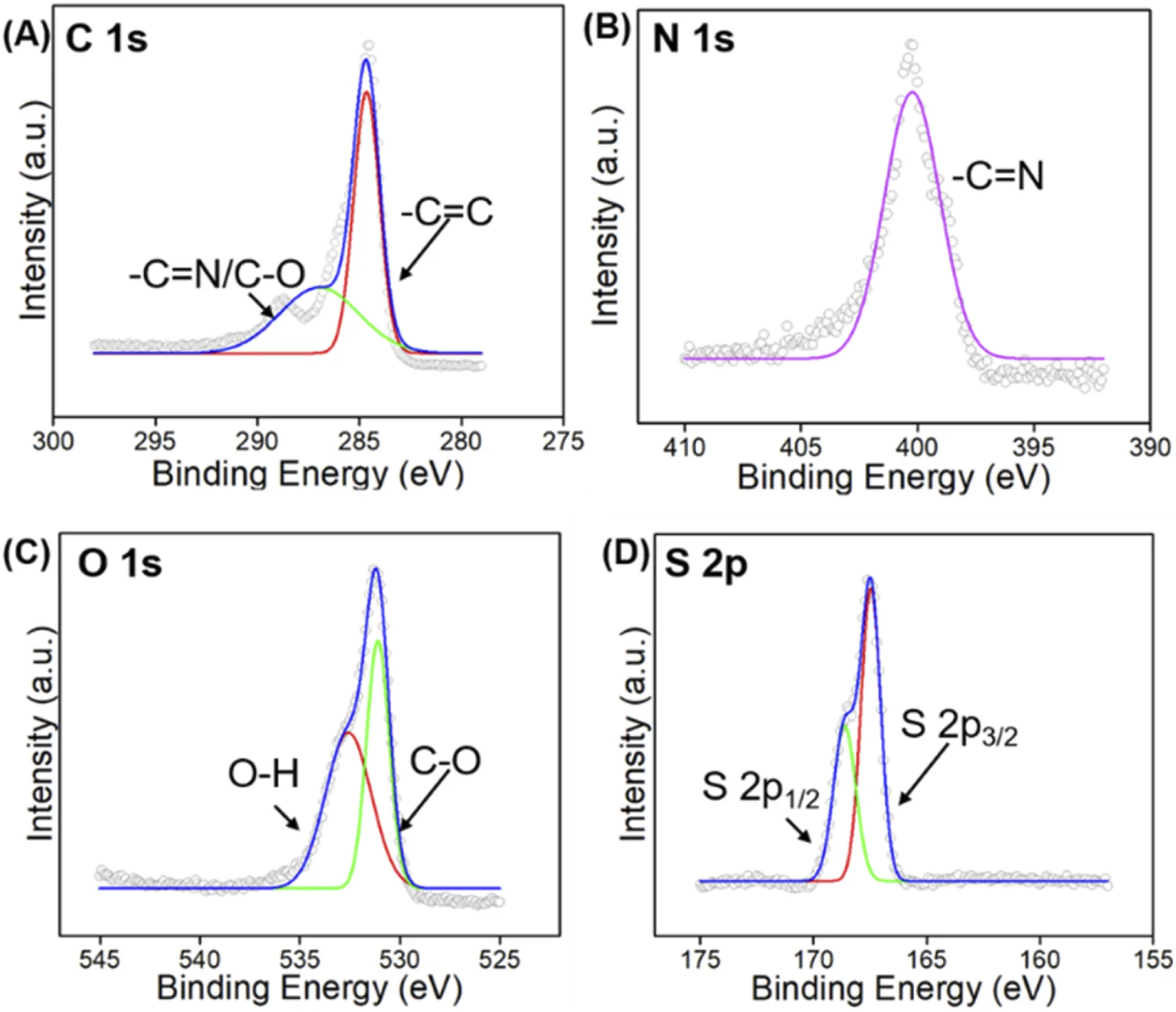
Deconvoluted XPS spectra of COF–SO_3_H (A) C 1s (B) N 1s (C) O 1s (D) S 2p.

### Esterification of biomass-derived carboxylic acids

The catalytic activity of COF–SO_3_H can be understood based on the contribution of its Brønsted acidic and porous structure. The high acid-site density with *H*_0_ of 1.56 suggests that highly acidic sites are linked with the incorporated –SO_3_H groups. The material also has a BET surface area of 185 m^2^ g^−1^, a pore volume of 0.28 cm^3^ g^−1^ and an average pore diameter of ∼6.1 nm. The hierarchical pore structure and interconnected sheet-like morphology are supposed to allow diffusion of the carboxylic acid and alcohol molecules, and to enhance their accessibility to the acid sites. So, the catalytic activity is not just determined by the intrinsic acidity of the –SO_3_H groups, but also by the acid strength, acid-site density and accessibility in the porous COF structure. The esterification of biomass-derived carboxylic acids, *i.e.*, LA and SA, with *n*-butanol was carried out to obtain *n*-butyl levulinate (NBL) and succinate esters as products using COF–SO_3_H as a catalyst. The reaction parameters, such as catalyst amount, reaction time, molar ratio (acid to alcohol), and temperature, were studied to achieve optimal conversion and selectivity of the desired product.

### Esterification of LA

The effect of the catalyst loading on the yields of LA esterification clearly shows the contribution of accessible acidic sites. The LA conversion was found to be significantly improved from 65% to around 96% with an increase in the amount of the catalyst from 10 to 40 mg, and the selectivity towards the formation of *n*-butyl levulinate increased up to 99.7% (Fig. 6A). This improvement is due to the enhancement in the amount of available –SO_3_H sites as the catalyst loading was increased. The –SO_3_H groups act as Brønsted acid sites, which allow protonation of the carboxylic carbonyl group, making it more electrophilic and allowing nucleophilic attack by *n*-butanol. Therefore, the more prevalent the accessible acid sites, the more often there will be productive interactions between acid and substrate. Based on the acid-site density measured *i.e.* 5.69 mmol g^−1^, the amount of acid functionality introduced to the reaction medium at 40 mg loading is ∼0.228 mmol. The conversion did not significantly improve with increasing amount of catalyst, indicating that, under the conditions of the reaction, the available amount of substrate and the mass-transfer characteristics are limiting once enough acid sites are available for the reaction. Hence, an optimum loading of the catalyst (40 mg) was chosen. Reaction time plays a crucial role in driving the reaction toward completion (Fig. 6B). At 6 h, moderate conversion, *i.e.*, 56% was obtained, with high selectivity of 92.5%. Increasing the reaction time to 8 h yields a 96% conversion with 99.7% selectivity towards *N*-butyl levulinate. Further extension to 10–12 h does not significantly improve conversion or selectivity, confirming that the reaction reaches equilibrium around 8 h. Therefore, 8 h is sufficient to achieve maximum efficiency, avoiding unnecessary energy consumption and prolonged operation.

**Fig. 6:**
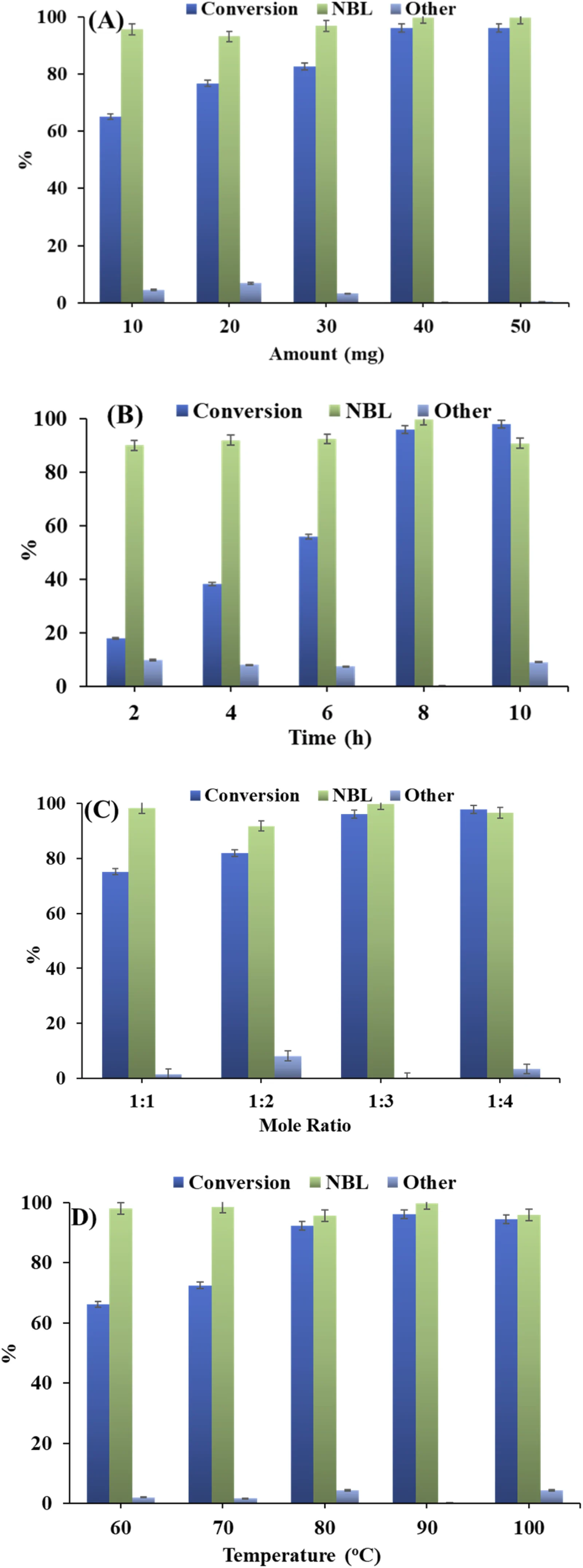
(A) Effect of amount; LA (10 mmol), *n*-butanol (30 mmol), time (8 h), temperature (90 °C); (B) effect of time; LA (10 mmol), *n*-butanol (30 mmol), amount (40 mg), temperature (90 °C); (C) effect of mole ratio; amount (40 mg), time (8 h), temperature (90 °C); (D) effect of temperature; LA (10 mmol), *n*-butanol (30 mmol), amount (40 mg), temperature (90 °C).

The reactant mole ratio significantly affects the equilibrium and product distribution (Fig. 6C). At a 1 : 1 ratio, the conversion is 75%, although selectivity remains high. Increasing the *n*-butanol content shifts the reaction equilibrium toward ester formation, leading to a steady increase in conversion. Optimal performance is observed at a 1 : 3 molar ratio, where conversion reaches 96% and selectivity peaks at 99.7%, with negligible by-product formation. The increased conversion can be attributed to both the greater availability of the alcohol nucleophile at the accessible acid sites, and the thermodynamic driving force of the esterification equilibrium. Still, an increase of alcohol ratio to 1 : 4 was not significant and slightly increased the amount of side products. This suggests that, beyond an optimum level, increasing alcohol concentration dilutes the substrate and may promote competitive adsorption, reducing productive acid–substrate interactions. Thus, the optimum condition was chosen as the 1 : 3 molar ratio. Temperature strongly influences reaction kinetics (Fig. 6D). At temperatures around 60–70 °C, conversion remains limited to 66–72% despite high selectivity, indicating insufficient activation energy for efficient hydrogenation. Raising the temperature to 80 °C significantly boosts conversion to 92%, while a further increase to 90 °C gives the highest conversion of 96% and 99.7% selectivity with minimal by-products. The temperature dependence suggests that the elementary steps that involve adsorption, activation of carbonyl, nucleophilic attack and elimination of water are easier at higher temperatures. However, 90 °C was chosen as the optimum temperature because it achieved good conversion and selectivity. Hence, 90 °C is identified as the optimal reaction temperature.

To obtain a maximum conversion 96% with 99.7% selectivity, the optimised conditions were determined as 40 mg catalyst, 8 h reaction time, 1 : 3 LA : *n*-butanol ratio and 90 °C reaction temperature.

### Esterification of SA

Similar to LA, the esterification of SA to monobutyl succinate and dibutyl succinate was also carried out under varying reaction conditions, and the results are shown in Fig. 7. In order to investigate the effect of the amount of catalyst, the esterification of SA was performed with different amounts of catalyst, *i.e.* 10, 20, 30, 40, 50 mg, and the results are presented in Fig. 7A. Increasing the catalyst amount from 10 to 40 mg resulted in a significant increase in conversion. In other words, as the concentration of COF–SO_3_H increases, the number of Brønsted acid sites also increases, and thus the probability of the first and second esterification steps increases. Further, it is worth noting that at lower catalyst amounts, the monoester product was predominant, whereas a higher catalyst loading leads to further esterification of monoesters due to the presence of a higher density of –SO_3_H groups.

**Fig. 7:**
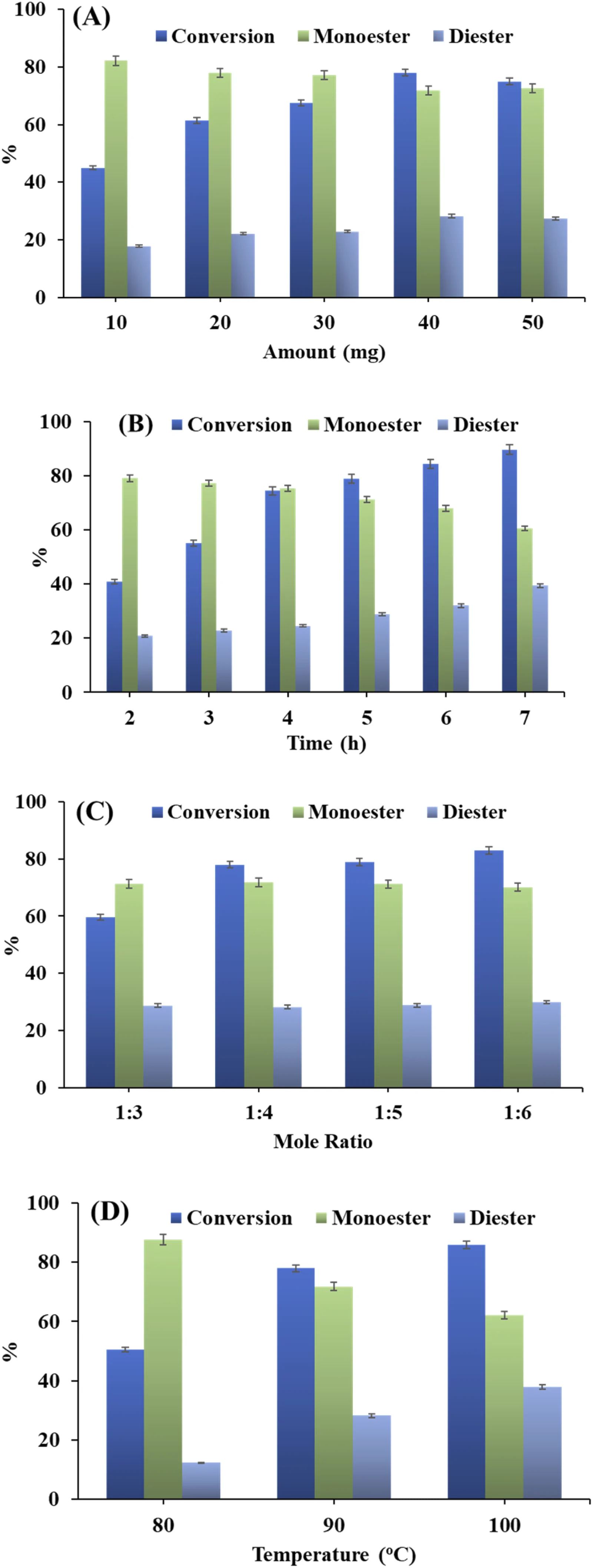
(A) Effect of amount; SA (10 mmol), *n*-butanol (40 mmol), time (5 h), temperature (90 °C); (B) effect of time; SA (10 mmol), *n*-butanol (40 mmol), amount (40 mg), temperature (90 °C); (C) effect of mole ratio; amount (40 mg), time (5 h), temperature (90 °C); (D) effect of temperature; SA (10 mmol), *n*-butanol (40 mmol), amount (40 mg), temperature (90 °C).

A similar trend in conversion and selectivity was observed when the reaction was monitored over time. As seen in the Fig. 7B, conversion and selectivity increase significantly with reaction time. An increase in reaction time from 4 to 7 led to an improvement in conversion from 66% to 86%, accompanied by a rise in diester selectivity from 23% to 36% and a corresponding decline in monoester selectivity from 76% to 63%, suggesting further esterification to the thermodynamically more stable diester. Furthermore, according to Le Chatelier's principle, upon increasing the molar ratio of *n*-butanol from 1 : 3 to 1 : 6 (Fig. 7C) significantly boosted overall conversion and shifted product selectivity towards diester formation because excess alcohol accelerates the equilibrium and increases the possibility of double esterification.^28^ Under these conditions, monoester selectivity gradually decreases highlighting its role as an important intermediate rather than a final product. Thus, the result reveals the synergistic interaction between catalytic site density, reaction kinetics, and equilibrium in COF-mediated biomass valorization processes by demonstrating that the selective formation of mono-*versus* diester can be effectively controlled by modifying reaction parameters. Higher catalyst loading, prolonged reaction times, and excess alcohol favour complete esterification to diester. Therefore, 1 : 4 was optimized as it showed desirable conversion and selectivity (*i.e.* 78% conversion, ∼71% monoester and ∼28% diester) with respect to others.

Among the evaluated parameters, reaction temperature plays a key role in shifting the equilibrium of the reversible reaction toward the forward direction. The temperature effect was examined between 80 and 100 °C shown in Fig. 7D. As expected, increasing the temperature from 80 to 100 °C resulted in significant improvements in both conversion and diester selectivity, increasing from 52% to 85% and from 11% to 37%, respectively. This behaviour can be attributed to increased molecular collisions at elevated temperatures, and since the reaction is endothermic, elevated temperatures favour esterification. Hence, temperature is one of the most critical factors in the overall optimisation process. Hence, 90 °C was selected as the ideal temperature for optimising reaction temperature.

### Substrate scope

It is essential to demonstrate the versatile activity of the catalyst through substrate studies. As shown in Fig. 8A, the alcohol chain length and structure have significant influence on conversion and selectivity. Methanol exhibited a moderate conversion of 72% with excellent selectivity, in contrast to ethanol and propanol. With an increase in alcohol chain length and branched alcohols, the selectivity for the desired ester is achieved, as side reactions are supposedly suppressed. Among alcohols, butanol showed the highest conversion of 96% with 99.7% selectivity. The catalyst scope was further explored for the esterification reaction of SA using different alcohols and the results are presented in Fig. 8B. Among the alcohols used, linear chain alcohols (*i.e.*, methanol, ethanol, *n*-propanol, *n*-butanol and *n*-hexanol) gave moderate to high conversion and comparatively lower fractions of diester. The higher diester formation observed for small-chain alcohols such as methanol and ethanol is due to their lower steric hindrance, which enhances nucleophilic attack on both the –COOH groups of succinic acid. As the carbon chain increases, conversion remains relatively high, nevertheless, a steady decline in diester formation is noted, which may be due to increased steric hindrance and reduced accessibility of the second –COOH group. In contrast, in branched alcohol, iso-propanol depicts low conversion, whereas iso butanol depicts moderate conversion with negligible diester formation. Similarly, a trend observed for secondary alcohol (*i.e.* sec-butanol) shows low conversion with negligible diester formation. This may be due to steric hindrance around the hydroxyl group and reduced nucleophilicity, which restrict the conversion of alcohol to the carbonyl carbon of succinic acid. Concluding, the esterification of succinic acid is strongly affected by the carbon chains of various alcohols, where smaller and less hindered alcohols favour high conversion and diester formation, whereas branched and secondary alcohol elevates monoester selectivity. This illustrates the vital role of steric and electronic effects in regulating both reactivity and product distribution in the esterification process.

**Fig. 8:**
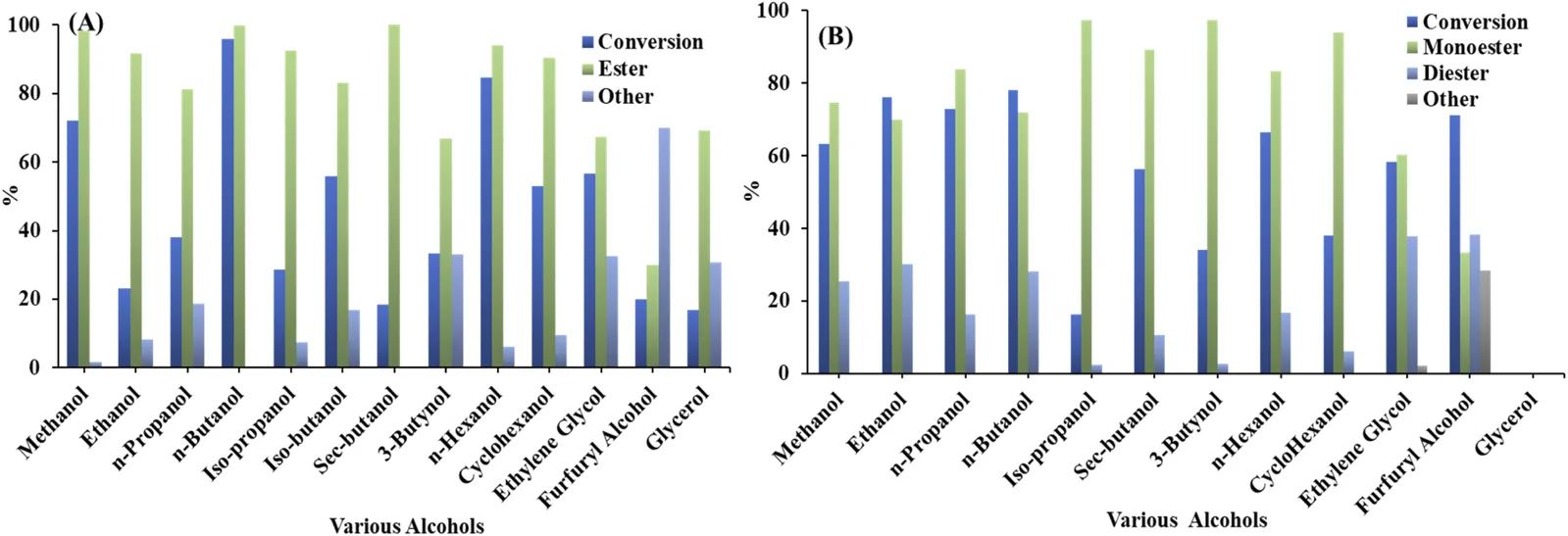
(A) Reaction condition; amount (40 mg), LA (10 mmol), alcohols (30 mmol), temperature (90 °C), time (8 h); (B) reaction condition; amount (40 mg), SA (10 mmol), alcohols (40 mmol), temperature (90 °C), time (5 h).

The substrate scope was extended further to several multifunctional and biomass-based alcohols such as ethylene glycol, glycerol and furfuryl alcohol. The acidic COF was not only found to be able to accept a diol substrate (*e.g.*, ethylene glycol) for conversion, but also to react with it in the form of ester. Glycerol showed significantly less conversion than *n*-butanol, which has three hydroxyl groups. The lower overall conversion may be related to the greater functionality and complexity of glycerol, which could result in several competitive interactions with the acidic sites and the formation of a range of products. More importantly, the formation of esters has been observed, showing the potential of the COF–SO_3_H active sites to catalyze esterification of the polyhydroxylated substrates. Another biomass-based alcohol, furfuryl alcohol, demonstrated decreased conversion and a different product distribution when compared to *n*-butanol. In addition to steric effects, the electron density and structure of the alcohol also play a role in its interaction with the acidic porous environment. The furan ring and the primary hydroxymethyl group are structurally unique features of the substrate as compared to other alcohols and could affect how accessible the reactive hydroxyl group is and the subsequent esterification pathway *via* the COF structure. However, ester products are formed, demonstrating that COF–SO_3_H can be used for structurally diverse alcohol substrates. Similarly, in the case of SA esterification with biomass-based alcohols, ethylene glycol and furfuryl alcohol show that the COF–SO_3_H is capable of catalyzing these alcohols with moderate conversion. However, esterification of SA with glycerol does not work at all due to the steric effect of both SA and glycerol.

### Possible mechanism of esterification

The esterification of LA with *n*-propanol over COF–SO_3_H is proposed to proceed mainly through a Brønsted acid-assisted Fischer esterification mechanism.^29^ The sulfonic acid groups anchored within the COF framework act as proton-donating active sites, while the porous structure of the catalyst facilitates the diffusion and contact of LA and *n*-butanol with these acidic centers (Fig. 9). In the first step, LA is adsorbed on the COF–SO_3_H surface through interactions with the acidic –SO_3_H sites, followed by protonation of the carboxylic carbonyl oxygen. This protonation increases the electrophilicity of the carbonyl carbon, making it more susceptible to nucleophilic attack by the oxygen atom of *n*-butanol. The attack of *n*-butanol leads to the formation of a tetrahedral oxonium intermediate, which subsequently undergoes proton transfer to convert the hydroxyl group into a better leaving group. The elimination of water then occurs, accompanied by re-formation of the carbonyl group, producing a protonated ester intermediate. Finally, deprotonation releases *n*-butyl levulinate as the desired ester product and regenerates the COF–SO_3_H active site for the next catalytic cycle. This pathway is consistent with the commonly reported acid-catalyzed esterification of levulinic acid, where Brønsted acid sites promote carbonyl activation, alcohol addition, dehydration, and catalyst regeneration.

**Fig. 9:**
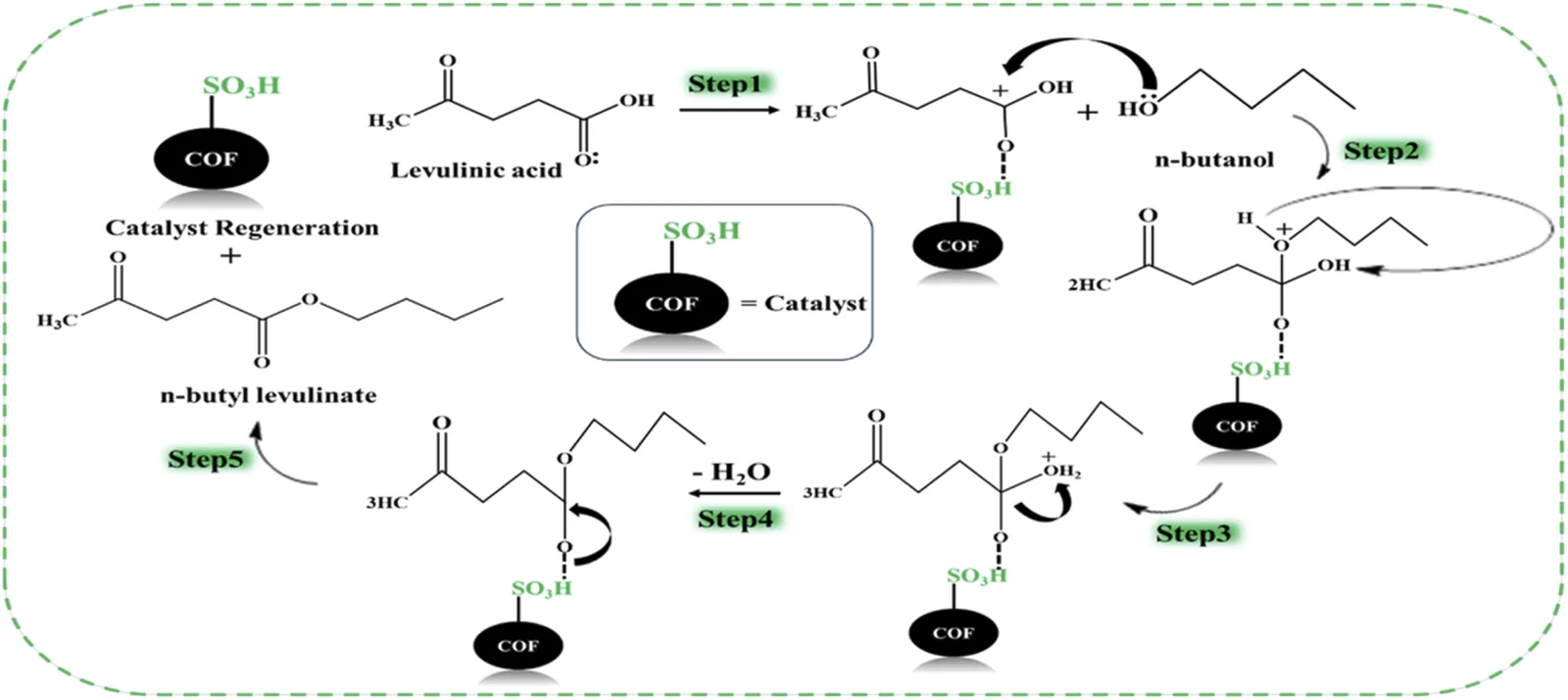
Proposed mechanism for esterification of LA with *n*-butanol.

The esterification of SA with *n*-butanol over COF–SO_3_H is proposed to proceed through a Brønsted acid-assisted surface mechanism involving consecutive monoester and diester formation. Initially, one carboxylic group of succinic acid is adsorbed within the porous COF framework through hydrogen-bonding interactions with the sulfonic acid and phenolic functionalities (Fig. 10). The –SO_3_H groups act as Brønsted acidic sites and protonate the carbonyl oxygen of the adsorbed carboxylic group, thereby increasing the electrophilicity of the carbonyl carbon. Subsequently, the oxygen atom of *n*-butanol attacks the activated carbonyl carbon to form a tetrahedral oxonium intermediate. After proton transfer, elimination of water occurs, followed by deprotonation to regenerate the COF–SO_3_H active site and release mono-butyl succinate. Since COOH group in monobutyl succinate can undergo the same acid-catalyzed sequence to form dibutyl succinate. Therefore, the final monoester/diester distribution is governed by the availability of accessible Brønsted acid sites, alcohol concentration, reaction time, temperature, and steric accessibility of the second carboxylic group. In the present COF–SO_3_H system, the predominance of monobutyl succinate under the optimized conditions suggests that the first esterification step proceeds more readily, whereas further conversion to dibutyl succinate is limited by equilibrium, steric effects of *n*-butanol, and the accessibility of the second –COOH group within the catalytic environment. This interpretation is consistent with reported succinic acid esterification mechanisms, where stronger or more abundant Brønsted acidic sites promote higher diester formation, while restricted acid-site availability or competitive adsorption favors monoester selectivity.

**Fig. 10:**
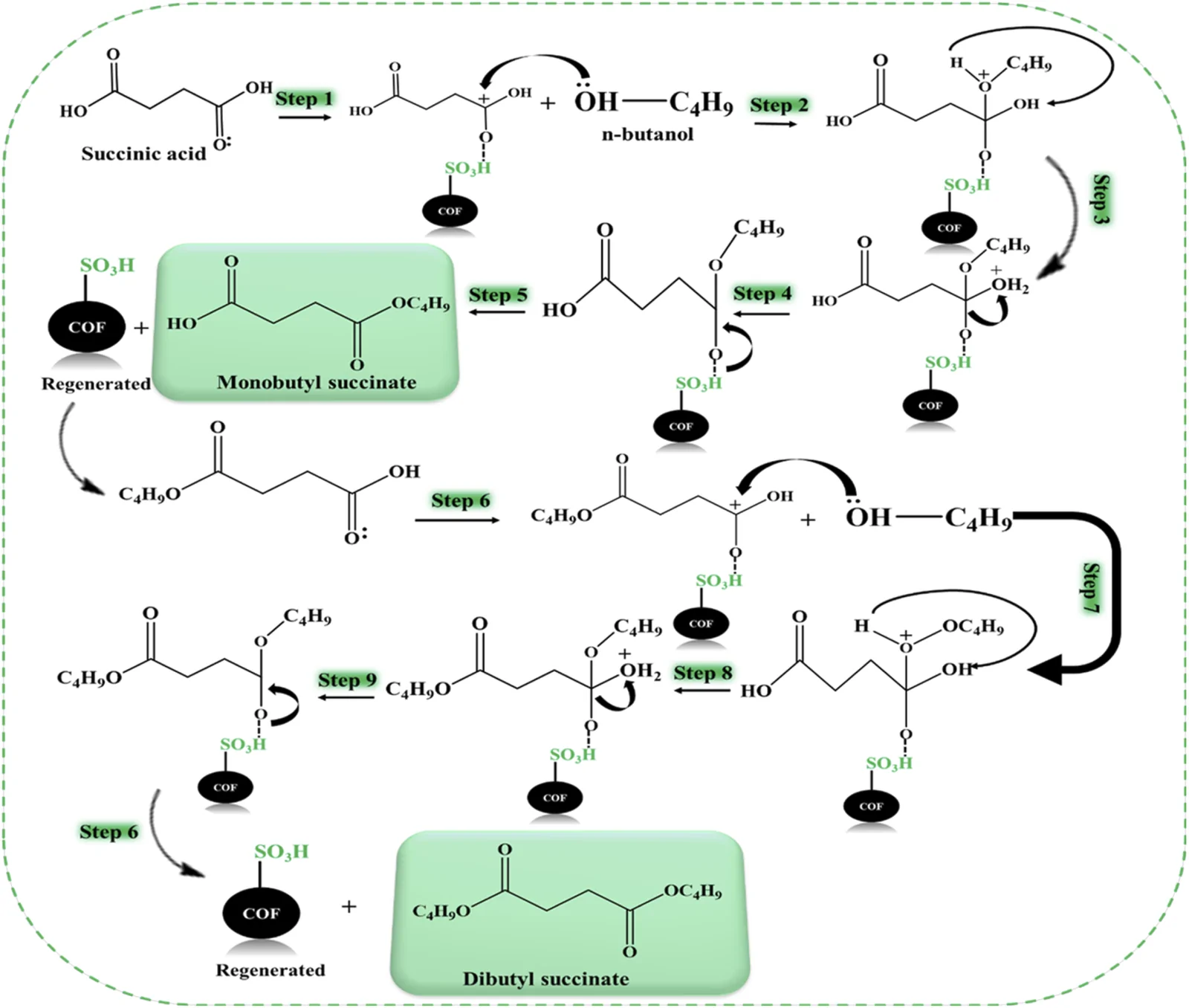
Proposed mechanism for esterification of SA with *n*-butanol.

### Heterogeneity test

The heterogeneity test was carried out for the esterification of LA and SA with *n*-butanol under optimized conditions. To prove COF–SO_3_H catalyst is heterogeneous, the test was carried out by filtering the catalyst from the reaction mixture (at 90 °C) after 6 h for LA and 4 h for SA esterification reactions, and the filtrate was allowed to react for the remaining time (Fig. 11). The reaction mixture and the filtrate were analyzed by Gas Chromatography. The results show that no significant change in percentage conversion and selectivity was observed. Indicating that the COF–SO_3_H catalyst falls into category C *i.e.*, the active species does not leach and the catalyst is heterogeneous. This was further proved with ICP-OES analysis of the reaction mixture, which showed that no sulfur content was found in the reaction mixture.

**Fig. 11:**
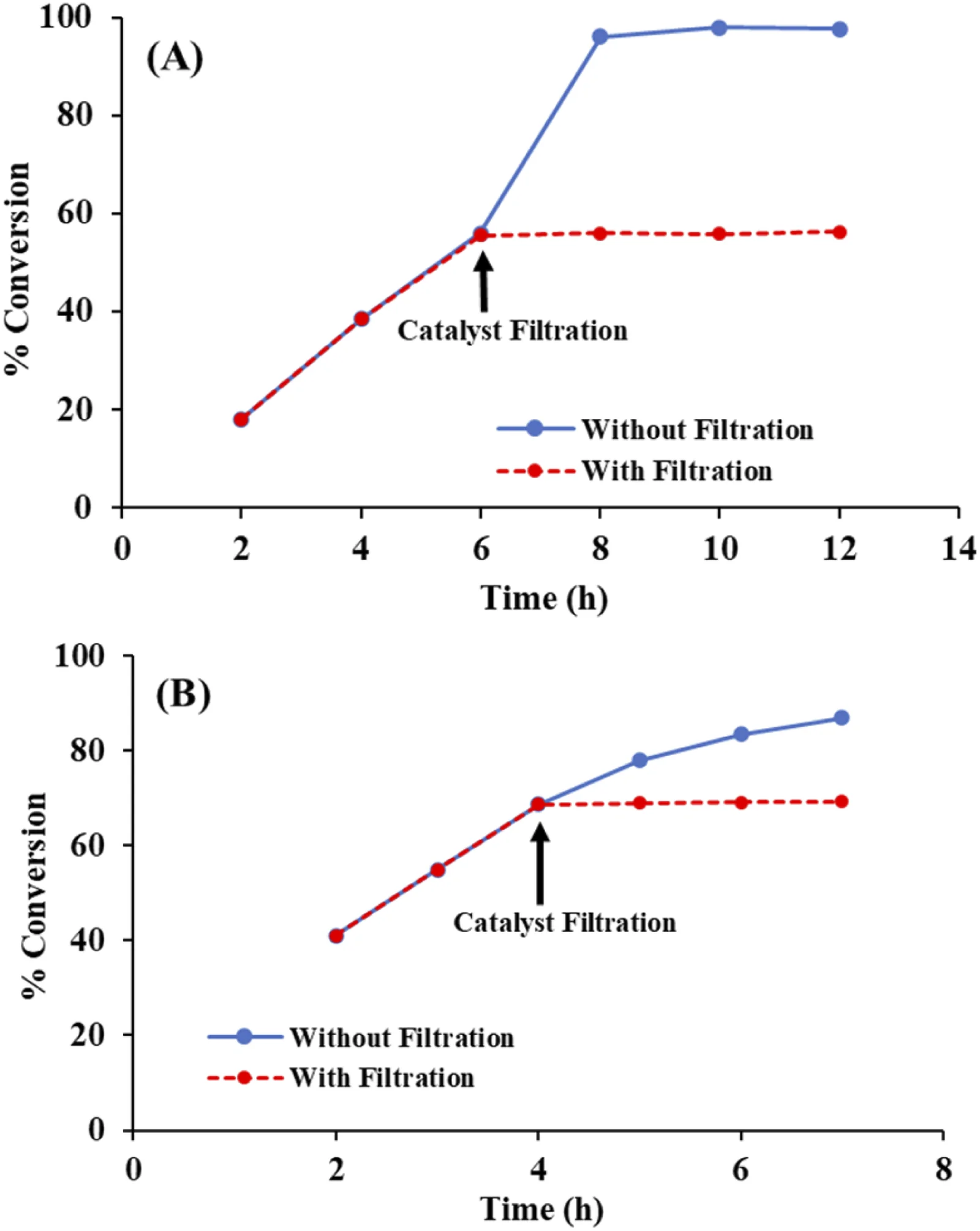
(A) LA (10 mmol), *n*-butanol (30 mmol), amount (40 mg), temperature (90 °C); (B) SA (10 mmol), *n*-butanol (40 mmol), amount (40 mg), temperature (90 °C).

### Regeneration, recycling of the catalyst and reaction scale-up

Recycling of the catalyst was performed for six successive cycles for levulinic acid esterification shown in Fig. 12A and five successive cycles for succinic acid esterification shown in Fig. 12B. The results showed no significant changes in conversion for levulinic acid esterification up to 5 cycles and for succinic acid esterification up to 4 cycles. It may be possible for further cycles, but we carried out only six cycles in levulinic acid esterification and only five cycles in succinic acid esterification. The catalyst was regenerated by centrifugation followed by methanol washing, and the regenerated catalyst (5th cycle) was characterised using SEM, FTIR and XPS (Fig. 13). The SEM and FTIR data suggest slight changes in morphology and chemical composition, which may account for the minor changes in conversion and selectivity. The XPS spectrum of the recycled COF–SO_3_H catalyst exhibited the same characteristic peaks as the fresh catalyst, corresponding to C 1s, N 1s, O 1s, and S 2p signals. The retention of the C=N, C–O, O–H, and S=O confirming that the imine-linked framework and pendant –SO_3_H groups remained intact after recycling. No significant peak shifts or additional signals were observed, indicating the absence of notable structural degradation or chemical transformation during the catalytic cycles. These results demonstrate the excellent chemical stability and recyclability of the synthesized COF–SO_3_H catalyst.

**Fig. 12:**
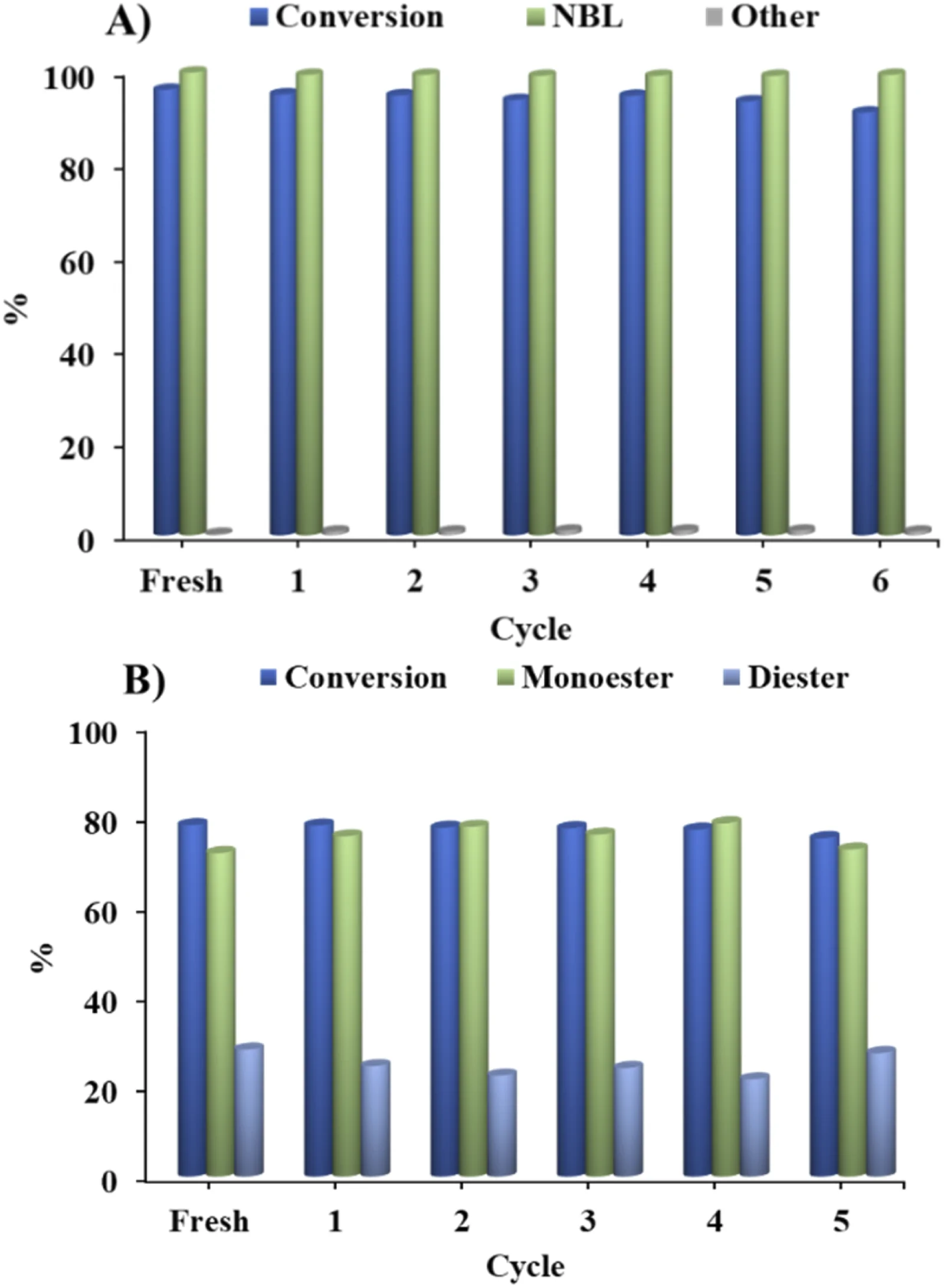
(A) Recycled catalyst; LA (10 mmol), *n*-butanol (30 mmol), amount (40 mg), temperature (90 °C), time (8 h); (B) recycled catalyst; SA (10 mmol), *n*-butanol (40 mmol), amount (40 mg), temperature (90 °C), time (5 h).

**Fig. 13:**
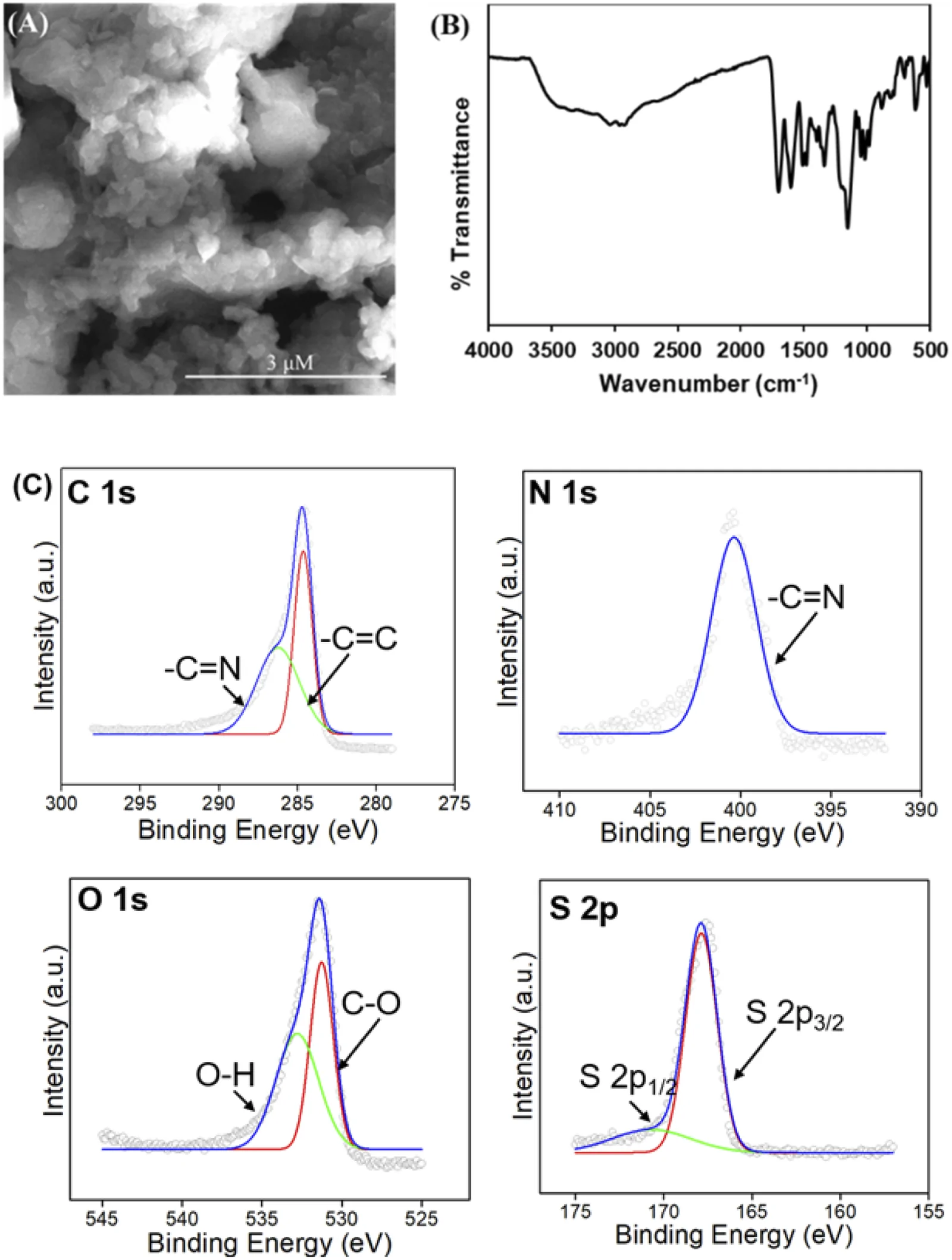
(A) SEM, (B) FTIR and (C) XPS of recycled catalyst after 5 cycles.

To explore industrial applicability, the esterification of LA and SA was carried out by scaling up the reactants 5-fold. The scale-up data showed comparative conversion (95% LA and 76% for SA) with a negligible decrease in selectivity for the products, suggesting the current catalytic systems can be implemented at large-scale ester production. However, further scale-up may involve deeper studies and process optimization with respect to thermodynamic and kinetic parameters.

### Kinetic studies of esterification reaction over COF–SO_3_H catalyst

The kinetic studies of LA and SA esterification with *n*-butanol were performed for 2–7 h and 2–6 h reaction times at 90 °C (Fig. S2). The LA conversion at each reaction time was determined experimentally and the concentration of unreacted LA was calculated. Similarly, the concentration of unreacted *n*-butanol was calculated from the stoichiometric relationship. The obtained data were analyzed for both first- and second-order kinetic models. The first-order model provided a correlation coefficient of (*R*^2^ = 0.966), with an apparent rate constant of 3.1 × 10^−3^ min^−1^. The reactants were initially not in equal concentrations, and so the appropriate unequal-reactant second-order expression was also tested. This model gave a relatively lower *R*^2^ = 0.956 as compared to the first-order expression. Thus, the kinetic analysis suggests that the apparent pseudo-first-order kinetics with respect to LA are predominant under the reaction conditions investigated.

At the same time, the plot of ln(*A*_0_/*A*) *versus* reaction time showed excellent linearity (*R*^2^ = 0.9976) for the succinic acid esterification process and indicated apparent pseudo-first-order kinetic behaviour of succinic acid. For the second-order kinetic model with unequal-reactant order, excellent linearity (*R*^2^ = 0.9970) was obtained. However, the correlation obtained with the non-equal-reactant second-order model suggests that the present data are not enough to determine the order of the reactions and require deeper kinetic studies for succinic acid esterification over COF–SO_3_H. It is important to calculate the activation energy for a particular system to check its validity for the chemical step. The activation energy for LA and SA esterification was 39.7 kJ mol^−1^ and 56.0 kJ mol^−1^, respectively. It is known that reactions with >25 kJ mol^−1^ activation energy are governed by a chemical step.^30^ For both LA and SA esterification reactions, the activation energy is higher than 25 kJ mol^−1^, which confirms that the reactions are truly governed by a chemical step.

### Comparison with the reported catalyst

Table 1 evaluates the comparison data of levulinic acid esterification for COF–SO_3_H catalyst with previously reported systems.^10,22,31–35^ Most of the reported catalysts exhibit comparable or better turnover number (TON) and turnover frequency (TOF). However, in contrast to these reported systems based on metal oxides, heteropolyacids, and metal salts, the present catalyst is completely metal-free, thereby eliminating issues associated with metal leaching, toxicity, and secondary environmental contamination. The porous and highly tunable COF framework provides abundant accessible acidic sites that facilitate efficient esterification performance. Moreover, reported catalysts require harsher temperatures, high catalyst loading, microwave assistance, or pressurized conditions to achieve comparable activity. In comparison, the synthesized COF–SO_3_H catalyst achieved 96% conversion and 99.7% selectivity toward *n*-butyl levulinate under comparatively moderate conditions (90 °C, 8 h, 1 : 3 LA : alcohol ratio), indicating the effective role of the porous COF framework and accessible –SO_3_H active sites in promoting esterification. A similar trend was observed for succinic acid esterification, as presented in Table 2. Unlike many reported catalysts^4,11,36–39^ containing metal oxides or polyoxometalates, the present catalyst avoids issues related to metal leaching and secondary contamination. The porous COF framework provides accessible Brønsted acidic sites that promote effective catalytic activity. Although some catalysts show slightly higher conversions and TON/TOF, they often require harsher conditions or metal incorporation. In comparison, the synthesized COF–SO_3_H catalyst provided 78% conversion under mild reaction conditions (90 °C, 5 h, 1 : 4 acid : alcohol ratio), demonstrating that the sulfonated porous COF structure can efficiently catalyze succinic acid esterification without severe operating conditions.

**Table 1:** Comparison of different catalysts for esterification of LA

Sr. no	Catalyst/system	Alcohol	Condition (temp, time, ratio)	Conversion (%)	TON (TOF h^−1^)	Ref.
1	Montmorillonite K10 (clay)	C_2_H_5_OH	170 °C, 3.75 h, N_2_ 0.6 MPa	96.5%	51 (13.6)	10
2	Copper molybdate (Cu_3_(MoO_4_)_2_(OH)_2_)	CH_3_OH	100 °C, 4 h, 1 : 20 MeOH:LA, 5% cat.	99.3%	46 (11.6)	32
3	ZrO_2_-supported phosphotungstic acid (20-HPW/Zr)	C_2_H_5_OH	150 °C, 3 h, 1 : 17 LA : EtOH	97.3%	— (—)	31
4	Porous organic polymer (BPOP–SO_3_H)	*n*-C_4_H_9_OH, others	30 min, not specified	98%	22.1 (44.3)	22
5	TiO_2_ nanoparticles	*n*-C_4_H_9_OH	120 °C, 8 h, 8.6 wt% cat.	77.6%	77.3 (9.6)	35
6	H_4_SiW_12_O_40_ (heteropolyacid)	C_6_–C_10_ alcohols	Room temp, not specified	∼90% conversion	10.6 (1.3)	34
7	Al_2_(SO_4_)_3_ (metal salt, MW)	CH_3_OH	110 °C, 10 min, 20 mol% cat.	99.4%	43.2 (259)	33
8	COF–SO_3_H	*n*-C_4_H_9_OH	90 °C, 8 h, 1 : 3	96% conversion	42.2 (5.2)	This work

**Table 2:** Catalyst comparison for succinic acid esterification

Sr. no	Catalyst	Alcohol	Conditions (temp, time, acid : alcohol ratio)	Conversion (%)	TON (TOF h^−1^)	Ref.
1	Anderson-polyoxometalate	C_3_H_7_OH	113 °C, 9.6 h, 4.9 : 1	77%	64.7 (6.7)	4
2	H^+^-β zeolite beta	Phenol	110 °C, 6 h, 1 : 3	96%	62.7 (2.6)	36
3	SiW12-MCM-22 (12-tungstosilicic acid)	*n*-C_4_H_9_OH	90 °C, 10 h, 1 : 3	97%	1214 (121.4)	37
4	12-Tungstophosphoric acid/zeolite HY	*n*-C_4_H_9_OH	90 °C, 8 h, 1 : 3	92%	1777 (222)	11
5	DBSA, Nafion NR-50, Novozym 435	C_8_H_17_OH	—	93%	9.0 (1.1)	38
6	Halloysite (natural clay)	CH_3_OH, C_2_H_5_OH, *n*-C_3_H_7_OH, *n*-C_4_H_9_OH	170 °C, 24 h, 10 mol% catalyst	90%	9.5 (0.4)	39
7	COF–SO_3_H	*n*-C_4_H_9_OH	90 °C, 5 h, 1 : 4	78%	34.3 (6.8)	This work

## Conclusion

A sulphonic acid functionalized covalent organic framework, COF–SO_3_H was successfully synthesized *via* an ultrasound-assisted aqueous-phase Schiff base condensation under mild conditions. The material exhibited a hierarchically porous structure with a BET surface area of 185 m^2^ g^−1^, pore volume of 0.28 cm^3^ g^−1^, and an average pore diameter of 6.1 nm. The measured Hammett acidity (*H*_0_) of 1.56 and acid-site density of 5.69 mmol g^−1^ confirmed the presence of strong and abundant Brønsted acid sites. Structural characterization, including PXRD, FTIR, and solid-state ^13^C NMR, confirmed the formation of the crystalline imine-linked framework and successful incorporation of –SO_3_H functionalities. These structural and acidic characteristics collectively provide accessible catalytic sites within a porous environment, highlighting the potential of COF–SO_3_H as a metal-free platform for biomass-derived acid valorization. Under optimized conditions, COF–SO_3_H afforded 96% conversion of levulinic acid with 99.7% selectivity toward *n*-butyl levulinate within 8 h. Succinic acid afforded 78% conversion within 5 h, with 71% monoester and 28% diester selectivity. The pronounced influence of alcohol carbon-chain length and molecular structure on conversion and product distribution indicates that catalytic performance is governed not only by the intrinsic acidity of the material but also by substrate accessibility and interactions within the porous framework. Esterification with several other primary and secondary alcohols further demonstrated the applicability of COF–SO_3_H toward structurally diverse alcohol substrates. However, the catalyst was not found to be effective for polyols such as glycerol. Importantly, the catalytic system is scalable, catalyst retained its activity over six cycles for levulinic acid and five cycles for succinic acid, with negligible loss in performance, demonstrating its potential as a recyclable heterogeneous acid catalyst. The scientific significance of this work lies in demonstrating that the integration of a high density of strong Brønsted acid sites within an ordered, porous COF can provide a metal-free catalytic environment capable of combining high conversion, excellent ester selectivity, and recyclability. Nevertheless, the present study has limitations regarding the detailed understanding of substrate adsorption, active-site accessibility, and the molecular-level origin of the observed alcohol- and substrate-dependent selectivity. Although the catalytic results are consistent with an acid-catalyzed esterification pathway, further experimental and computational investigations of adsorption energetics, reaction intermediates, and transition states are required to establish the mechanistic aspects more conclusively. In addition, long-term stability studies and detailed evaluation of acid-site retention and framework integrity under prolonged reaction conditions would strengthen the assessment of catalyst durability. Future studies should therefore focus on systematically tuning the pore architecture, –SO_3_H loading, and local chemical environment of the COF to control substrate diffusion and product selectivity. Investigation of a broader range of biomass-derived acids and alcohols, together with continuous-flow operation, extended recyclability, and scale-up studies, would help evaluate the practical potential of this catalyst. COF–SO_3_H provides a promising and tunable metal-free platform for sustainable esterification and biomass valorization, while the proposed mechanistic and durability studies represent important directions for advancing its catalytic application.

## Author contributions

Shradhanjali Samal: investigation, formal analysis, methodology, writing, review & editing, visualization. Muskan Memon: investigation, formal analysis, methodology, visualization. Entesar Alhousin: writing, methodology and visualization. Soyeb Pathan: conceptualization, supervision, project administration, data curation, writing – review and editing. Sonal Thakore: conceptualization, supervision, project administration, data curation, writing – review and editing, funding acquisition.

## Conflicts of interest

There are no conflicts to declare.

## Supplementary Material

RA-016-D6RA06829D-s001

## Data Availability

The data supporting this article have been included as part of the supporting information (SI). Supplementary information is available. See DOI: https://doi.org/10.1039/d6ra06829d.
