# Supplementary material for: Biomass-to-biomass strategy: sulphonic acid functionalized covalent organic frameworks for metal-free esterification of biomass-derived acids
Source: RSC Adv. 2026 Oct 7;16(46):60681–95. doi: 10.1039/d6ra06829d (PMC13643848; doi:10.1039/d6ra06829d)
Supplement: Supplementary file 1 [file RA-016-D6RA06829D-s001.pdf]

Supplementary Information (SI) for RSC Advances.
This journal is © The Royal Society of Chemistry

Biomass-to-Biomass Strategy: A Sulphonated Covalent Organic Framework for Metal-Free Esterification of Biomass-Derived Acids

Shradhanjali Samal[a], Muskan Memon[b], Entesar Alhousin[a], Soyeb Pathan[b,c*], Sonal Thakore[a*]

[a] Department of Chemistry, Faculty of Science, The Maharaja Sayajirao University of Baroda, Vadodara 390 002, India

[b]Research and Development Cell (RDC), Parul University, Vadodara 391760, India.

[c]Department of Chemistry, Parul Institute of Applied Sciences, Parul University, Waghodia, Vadodara, Gujarat, 391760, India

*Author for correspondence E-mail: chemistry2797@yahoo.com , sonal.thakore-chem@msubaroda.ac.in (Thakore, S.), khan_9751@yahoo.com (Pathan, S.)

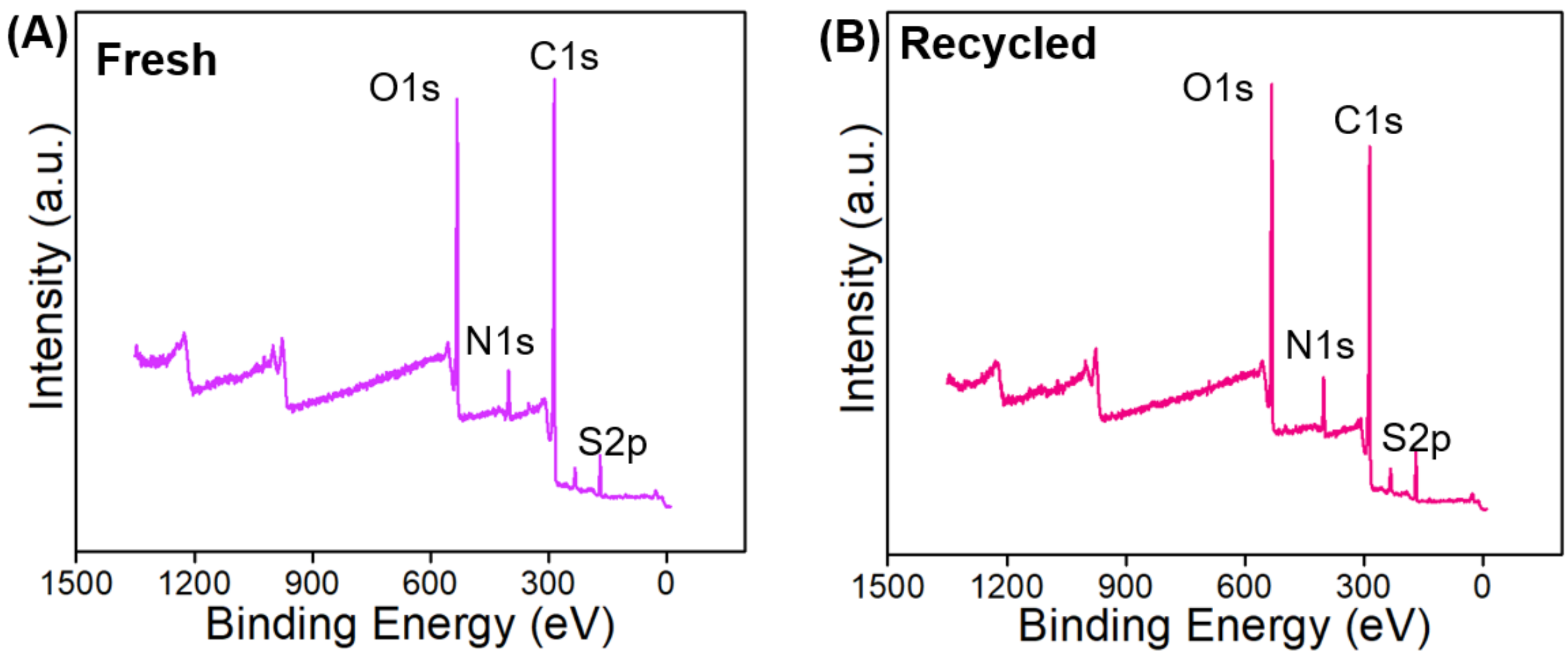

Figure S1. XPS survey spectra of **(A)** fresh and **(B)** recycled catalyst

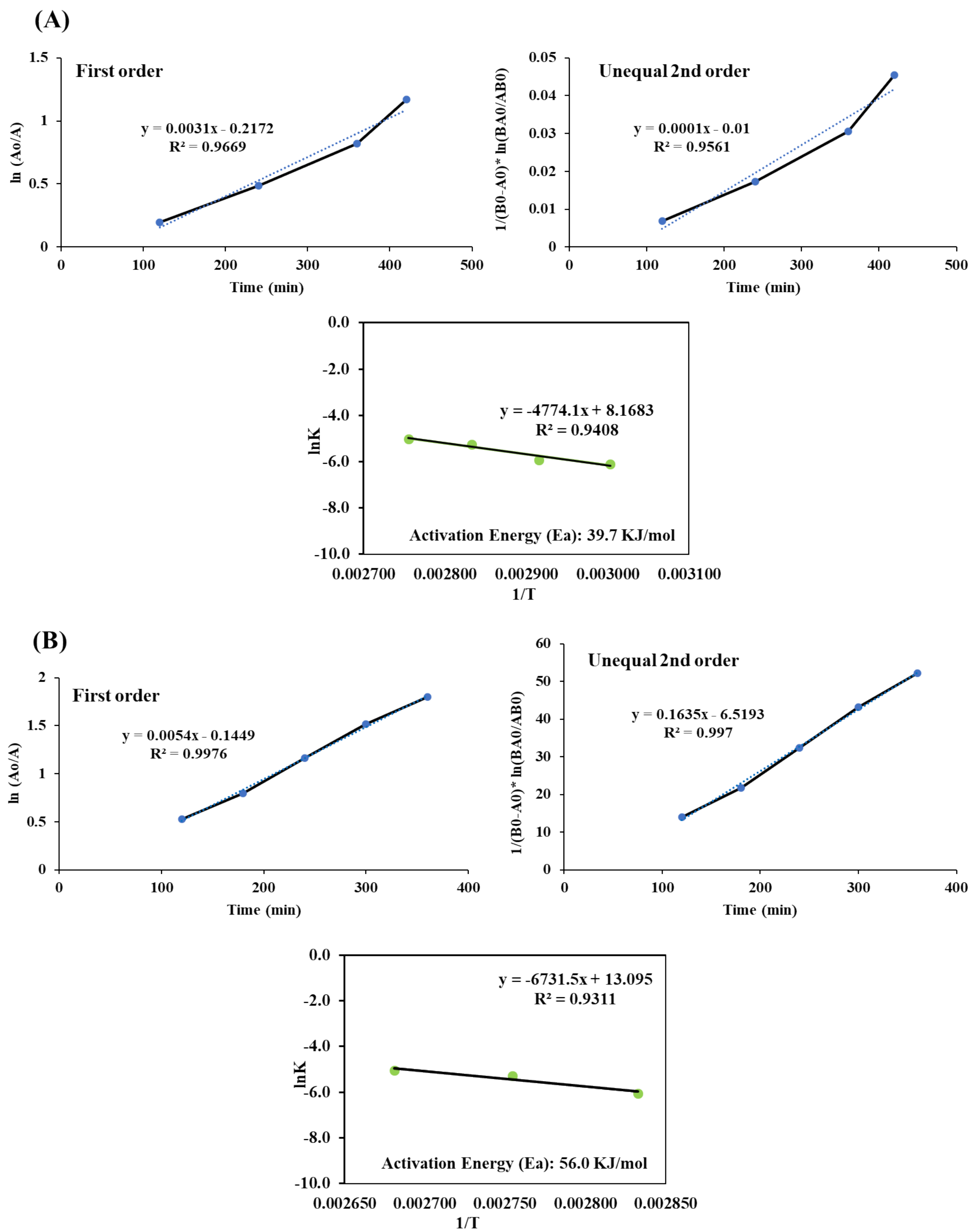

Figure S2. Kinetics of the esterification reaction of (A) LA and (B) SA with n-butanol.
